# An analytical model to minimize the latency in healthcare internet-of-things in fog computing environment

**DOI:** 10.1371/journal.pone.0224934

**Published:** 2019-11-13

**Authors:** Saurabh Shukla, Mohd Fadzil Hassan, Muhammad Khalid Khan, Low Tang Jung, Azlan Awang

**Affiliations:** 1 Centre for Research in Data Science (CeRDaS), Computer and Information Science Department, Universiti Teknologi PETRONAS(UTP), Seri Iskandar, Perak Darul Ridzuan, Malaysia; 2 College of Computing and Information Sciences, PAF-KIET, Karachi, Pakistan; 3 Electrical and Electronic Engineering Department, Universiti Teknologi PETRONAS(UTP), Seri Iskandar, Perak Darul Ridzuan, Malaysia; King Saud University, SAUDI ARABIA

## Abstract

Fog computing (FC) is an evolving computing technology that operates in a distributed environment. FC aims to bring cloud computing features close to edge devices. The approach is expected to fulfill the minimum latency requirement for healthcare Internet-of-Things (IoT) devices. Healthcare IoT devices generate various volumes of healthcare data. This large volume of data results in high data traffic that causes network congestion and high latency. An increase in round-trip time delay owing to large data transmission and large hop counts between IoTs and cloud servers render healthcare data meaningless and inadequate for end-users. Time-sensitive healthcare applications require real-time data. Traditional cloud servers cannot fulfill the minimum latency demands of healthcare IoT devices and end-users. Therefore, communication latency, computation latency, and network latency must be reduced for IoT data transmission. FC affords the storage, processing, and analysis of data from cloud computing to a network edge to reduce high latency. A novel solution for the abovementioned problem is proposed herein. It includes an analytical model and a hybrid fuzzy-based reinforcement learning algorithm in an FC environment. The aim is to reduce high latency among healthcare IoTs, end-users, and cloud servers. The proposed intelligent FC analytical model and algorithm use a fuzzy inference system combined with reinforcement learning and neural network evolution strategies for data packet allocation and selection in an IoT–FC environment. The approach is tested on simulators iFogSim (Net-Beans) and Spyder (Python). The obtained results indicated the better performance of the proposed approach compared with existing methods.

## Introduction

The latest report by the International Data Corporation stated that the number of Internet-related sensors will increase to 30 million by 2020, and the number of Internet-of-thing (IoT) devices will be in the range of 50 billion to 1 trillion [1]. Furthermore, U.S. factories will contain 500 million sensors, where 212 billion sensors will be available in the market [2]. Additionally, approximately 110 million cars will be connected to 5.5 billion sensors, while 1.2 million houses connected with 200 million sensors; 237.1 million wearable body devices are estimated to be available in the market by 2020 [3]. The worldwide IoT market is expected to reach $1.7 trillion in 2020. Additionally, 30.7% of IoT devices will be provided in the healthcare market. The healthcare market for IoTs is estimated to reach $117 billion by 2020 [2] with 507.5 zettabytes of data to be generated by 50 billion connected devices [4].

A large set of IoTs is currently being used in healthcare, which results in a large volume of data. To analyze, store, and pre-process the large variety, volume, and veracity of data, cloud servers are used worldwide. The cloud is currently the only available feasible solution for communications among healthcare IoTs [5]. Cloud computing ease the burden of healthcare IoT devices by removing battery-draining computational tasks [6, 7]. The cloud is the only place for the analysis, filtering, pre-processing, and aggregation of data generated from healthcare IoT devices. However, the cloud has its limitations concerning healthcare IoTs. Owing to the increasing transmission and the determination of these high volumes of data, the reaction time in cloud computing is increasing as well. An upsurge in reaction time results in a higher service latency to end-users. For large data transmissions, more data are transmitted over a network, hence the higher probability of an error occurring. Packet loss and transmission latency are proportional to the amount of data transmitted from IoTs to cloud servers. This causes a poor quality of service (QoS) to end-users. In many time-critical applications of the IoT, cloud-scale processing and storage are not required. Extreme time-bounded selections should be made closer to IoT devices. The healthcare infrastructure requires real-time data for time-sensitive applications. The critical requirements for healthcare IoTs are minimum latency and network bandwidth conservation [8]. The cloud and end-devices are connected via routers and gateways. Therefore, a large number of routers are placed between healthcare IoTs and the cloud. These routers incur computation delays. The larger the distance, the larger is the number of routers used between the source and destination. Data travel a long route from end-devices to a cloud server and consume a high bandwidth.

### Motivation

The main motivation for this study is the requirement of minimum latency with good QoS for time-critical healthcare IoT applications. The cloud cannot satisfy all these requirements. As a patient’s physiological state changes with time and to monitor remote patients, rapid decisions and agile responses are required. If network conditions are unpredictable, latency can become more uncertain. Owing to high latency, the patient health data (PHD) are not returned in real time. This render the data meaningless, inadequate, and unreliable. The situation worsens when the processing of cascading-based data is required (such as signal processing of electrocardiogram (ECG) or electroencephalogram (EEG) signals) [9, 10]. The delay of services in healthcare IoTs may vary from millisecond to microsecond [11, 12]. When the data size increases, the round-trip time delay for these healthcare IoT time-sensitive applications increase from milliseconds to seconds and from seconds to minutes [9, 11], thus worsening the real time operations of healthcare IoTs [13, 14]. See Table 1 shows the QoS requirements for medical data.

**Table 1:** QoS requirements for medical and healthcare data transfer rates [15, 16].

Healthcare services	Data rate	Delay	Packet loss percentage
Audio	4–25 kbps	150–400 milliseconds	3%
Video	32–384 kbps	150–400 milliseconds	1%
Electrocardiogram (ECG)	1–20 kbps	approx. 1 seconds	Zero
File Transfer	Not available	Not available	Zero

Table 2 shows the QoS requirements for E-healthcare services.

**Table 2:** QoS requirements for E-healthcare services [15, 16].

Services for E-healthcare	Healthcare applications	Type of media	Maximum delay
Real-time communication audio-based	Conferencing between patients/end-users and doctors (Audio)	Audio	< 150 milliseconds one-way
Real-time communication video-based	Conferencing between patients/end-users and doctors (Video)	Video	< 250 milliseconds one-way
Real-time robotic services	Remote based telesurgery	Robotic data, audio, and video	< 300 milliseconds round-trip-time
Real-time monitoring	Patient health data and vital sign transmission	Biomedical data collected by sensors	Application < 300 milliseconds for real-time ECG.

In January 2014, Cisco proposed a solution to address high latency and network bandwidth consumption between IoTs and the cloud by introducing the concept of fog computing (FC) to the world [17]. FC affords the features of the cloud to the edge of networks [18]. It acts at the edge of networks and. is a type of subcloud [19, 20]. It can be a gateway, router, laptop, or any device that serves as a middleware separating IoTs and the cloud. FC is proposed to reduce the burden of the cloud instead of replacing the cloud. The main goal of FC is to reduce the high latency between IoTs and the cloud. It has proximity to end-devices [21, 22].

**Fig 1:**
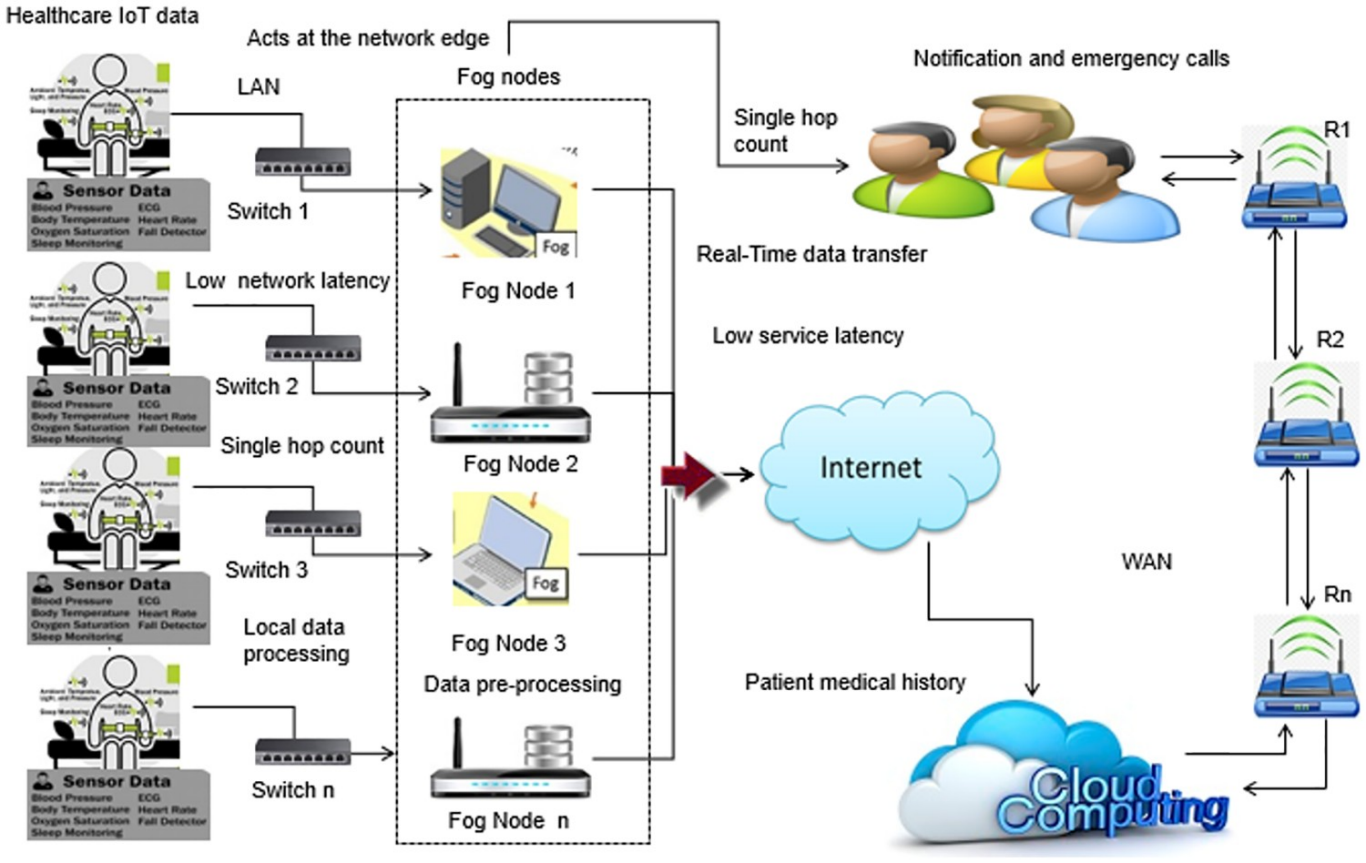
Data transmission between healthcare IoTs, end-users, and cloud servers using FC.

Fig 1 shows the transmission of healthcare data in real time to end-users. Here R_1_, R_2_, and R_n_ denote the number of routers used between end-users and cloud servers.

Hitherto, real-world implementations of FC are still rare; FC has primarily been mentioned in literature only [19, 23, 24]. Previous studies regarding FC were related to the standard approach of data communications among IoTs and the cloud; however, for the current scenario, an enhanced intelligent infrastructure is required to serve as a gateway between IoTs and the cloud. This intelligent gateway serves to obtain PHD in real time by reducing the computation, communication, and network latency between healthcare IoTs and cloud servers. Intelligent enhanced FC is a specialized and functional computing model that transfers healthcare data to end-nodes in real time.

### Contribution

The significant contributions of this study are as follows:

1. The FC-based analytical model is proposed to transfer healthcare IoT data in real time to end-users. The model allows fog nodes to determine the optimal functions to be conferred to a reward function. Hence, the fog node can serve as a controller to define its reward function based on the appropriate execution.
2. A novel hybrid machine learning algorithm is proposed, which uses the fuzzy inference system (FIS) and reinforcement learning (RL) technique based on neural network (NN) evolution strategies to address the problem of high latency between healthcare IoTs, end-users, and cloud servers. The healthcare IoT data is classified into low risk, normal, and high-risk using FIS. Next, the proposed algorithm uses RL and NN evolution strategies for the data packet allocation and selection in fog nodes. The proposed algorithm uses a unique approach and has a simpler data processing convolution and operation that is suitable for computers with parallel core CPUs. Recent research and algorithms in this area lack the hybrid machine learning approach to minimize high latency.
3. The proposed work reduces the total latency between healthcare IoTs and cloud servers. Here, the total latency (TL) is the sum of computation latency (*C*_*PL*_), communication latency (*C*_*L*_), and network latency (*N*_*L*_) i.e. TL = (*C*_*PL*_)+(*C*_*L*_)+(*N*_*L*_).

### Related work

This section presents an in-depth analysis and comparison between existing works and the present work, focusing on high latency, network usage, and bandwidth consumption in IoTs, and cloud and FC.

Wu *et al*. [25] discussed the requirements of information-centric social networks (ICSN); they applied fog computing security service (FCSS) in ICSN. The ICSN requirements are a deployment scheme, mobility of data, minimum latency, and effective end node communication. The use of fog computing in ICSN results in shifting computational tasks, resources, and intelligence from remote and distance servers to the edge of networks. FC-based content-aware filtering is used to secure the services in ICSN. However, the authors did not discuss the issue of high computational and network latency generated at the fog nodes. Dinh *et al*. [26] proposed a cost-effective deployment schema for services related to IoTs in fog and cloud networks. Their proposed schema measures the virtual network function (VNF) with the potential to improve the software function chaining (SFC) availability. The author discussed the issues related to hardware and software failure and resource limitations in FC nodes. Li *et al*. [27] proposed the service popularity-based smart-resources partitioning (SPSRP) method for IoTs and FC; they highlighted the issue of computing and resource efficiency on fog nodes. This proposed work seeks to reduce the delay and response times and fault tolerance in IoTs and fog servers. Similarly, Alam *et al*. [23] introduced the concept of a basic block offloading mechanism to deploy mobile codes on a geographically distributed fog mobile network in a decentralized manner. The RL technique was used to migrate the blocks in the distributed multi-agent environment. The result showed a reduction in high latency and processing time. Similarly, Kao *et al*. [28] proposed a new technique called Hermes to minimize latency in mobile computing for time-critical issues. The main function of the technique included the optimization of task assignments for devices that are deprived of resources. This technique was based on the offloading of computational tasks. Nishtala *et al*. [20] proposed a technique called Hipster to meet the demands of QoS in end-user requirements. The technique involved a combination of heuristic and RL. The combined machine learning effect managed the latency for time-sensitive workloads in cloud computing. However, the authors did not explain the issue of high communication latency in cloud servers. Naas *et al*. [29] highlighted the problem of high latency for time-critical IoT applications; to resolve it, they proposed a technique called iFogStor. This technique was based on the FC concept. In iFogStor, the issue of data placement was formulated as a generalized assignment problem (GAP). Furthermore, they recommended a method to solve the problem using accurate integer programming and a heuristic approach. This approach requires a more accurate model and architecture for time-sensitive IoT applications. Similarly, Pan *et al*. [30] discussed various emerging and existing technologies for IoT applications, such as cloudlet, edge-cloud, FC, and mobile-edge computing. They highlighted several existing issues related to IoT, such as high latency and data traffic. An internal study on existing and emerging technologies was conducted by the authors in their survey analysis. However, the research lacks consideration on practical implementation for latency minimization. Cao *et al*. [31] proposed a machine-learning algorithm to reduce the energy and bandwidth consumption and network usage for mobile devices. They discussed the issue of computational offloading of tasks for multiple users in the cloudlet environment. Next, Brogi *et al*. [32] proposed a model in the fog environment for IoT to support the QoS deployment infrastructure; they discussed several challenges, such as data distribution, segmentation, and adaptive deployment in IoT and the cloud infrastructure. However, Cao *et al*. [31] and Brogi *et al*. [32] did not explain the issues related to the high computational and network latency between IoT and cloud. Mahmud *et al*. [33] highlighted the problem of high latency and large data transmission in healthcare applications. Therefore, they proposed a cloud-fog based service along with a reference architecture for healthcare applications. An analysis of the obtained results was done with respect to data communication optimization, latency minimization, and reduction in power consumption. The results showed an improvement in cost efficiency, energy consumption, and network delay. Rafique *et al*. [34] proposed a hybrid bio-inspired algorithm to minimize the response and execution time in the IoT-fog-cloud environment. The hybrid algorithm was a combination of cat swarm and particle swarm optimization. The algorithm was modified to manage the availability of resources and task scheduling in fog nodes. Future work requires the use of the RL technique for resource management in the IoT-fog environment.

To protect and secure the data within the cloud environment, Ahsan *et al*. [35] proposed a centric FC-based scheme for cloud storage; they discussed several issues related to data security and privacy in cloud computing. The privacy of users’ data is of utmost importance in cloud servers. Therefore, a Xor combination was used to protect the data from unauthorized access and malicious attacks. The results were validated with respect to the data packet processing time. The authors used a new technique based on a hash algorithm to detect the data modification with maximum probability. Waqar *et al*. [36] proposed a framework to protect and secure the users’ data within the cloud from unauthorized intruders. The framework was based on a dynamic metadata and database schema design. Next, the dynamic metadata was reconstructed for privacy operations and applications. Different cryptographic operations were used to modify the database schema. Scope for future research includes the implementation of the proposed approach using RL techniques. Soleymani *et al*. [37] proposed a fuzzy-based model to collect correct and authorized information from vehicular ad hoc networks (VANETs). The vehicles in VANET require reliable information. Incorrect information would lead to interruption and system failure. The authors used fuzzy logic to make decisions for constructing rules related to the trust model in VANET. Their proposed work was based on the experience where distributed fog nodes were adopted to check event accuracy in VANETs.

The research analysis and comparison of techniques showed that the research to minimize the total latency (i.e. computational, communication, and network latency) between IoT and the cloud is incomplete. Therefore, a novel approach is required to minimize the high latency for time-sensitive applications in healthcare IoTs. A complete in-depth analysis and comparison of techniques used by different authors in their research are provided in Tables 3 and 4.

**Table 3:** The various techniques used by different authors in their proposed research works.

Techniques (*T*_*i*_(*i* = 1, 2, − − −*n*))
**T**_**1**_**:** Fog computing-based content-aware filtering and fog computing security service (FCSS) on edge networks [25].
**T**_**2**_**:** Cost-effective deployment schema for IoT using FC [26].
**T**_**3**_**:** Service popularity-based smart-resources partitioning (SPSRP) for IoT and FC nodes [27].
**T**_**4**_**:** RL algorithm and basic block offloading mechanism on fog mobile [23].
**T**_**5**_**:** Hermes: mobile computing [28].
**T**_**6**_**:** Hipster: heuristic and RL in cloud computing [20].
**T**_**7**_**:** iFogStor: heuristic approach in FC [29].
**T**_**8**_**:** Future edge cloud, cloudlet, FC and mobile edge computing [30].
**T**_**9**_**:** Computation offloading for multiple mobile users in a cloud computing environment [31].
**T**_**10**_**:** FC model for QoS deployment infrastructure in IoTs [32].
**T**_**11**_**:** Cloud-fog based services for healthcare [33].
**T**_**12:**_ A hybrid bio-inspired algorithm [34].
**T**_**13:**_ A fog centric cloud storage scheme [35].
**T**_**14**_**:** FC analytical model for healthcare IoTs using hybrid machine learning approach [Proposed Work].

**Table 4:** The comparative analysis for minimization of communication latency (*C*_*L*_), computation latency (*C*_*PL*_), and network latency (*N*_*L*_). The table also lists the authors’ names along with the techniques used.

Author	*T*_*i*_	(*C*_*L*_)	(*C*_*PL*_)	(*N*_*L*_)
Wu *et al*. [25]	T_1_	✓	X	X
Dinh *et al*.[26]	T_2_	✓	X	X
Li *et al*. [27]	T_3_	✓	X	X
Alam *et al*. [23]	T_4_	✓	✓	X
Kao *et al*. [28]	T_5_	✓	X	X
Nishtala *et al*. [20]	T_6_	X	✓	✓
Naas *et al*.[29]	T_7_	X	✓	✓
Pan *et*.*al*. [30]	T_8_	X	X	X
Cao *et al*. [31]	T_9_	✓	X	X
Brogi *et al*. [32]	T_10_	✓	X	X
Mahmud *et al*. [33]	T_11_	✓	X	✓
Rafique *et al*. [34]	T_12_	✓	✓	X
Ahsan *et al*. [35]	T_13_	X	✓	X
Proposed work	T_14_	✓	✓	✓

Different techniques used by authors in their research are selected as the baseline for comparison and analysis with our proposed approach. The discussed techniques work towards minimizing high latency, network consumption, and RAM consumption between IoTs and the cloud. These existing techniques mostly use conventional FC approaches as middleware gateways for data transmission between IoTs, end-users, and cloud servers. The mentioned techniques play a major role in healthcare IoTs. However, most of the existing works lack real-world implementation of latency minimization between IoTs, fog nodes, and cloud servers. Owing to the above-mentioned reasons, these existing techniques are selected for a comparative analysis with our proposed approach.

In this section, we identify the limitations of the existing techniques in the IoT-fog-cloud environment. In addition, the computation, network and communication latency and network usage are argued to be high and infeasible for healthcare IoTs. The issue of high latency in healthcare IoTs leads to delay in the transmission of PHD to end-users [2, 5]. The analysis of previous research shows that traditional cloud computing approaches and middleware gateways were unable to fulfill the latency demands and QoS requirements of healthcare IoTs [15, 16]. To date, no significant research has been done regarding healthcare IoTs to minimize the round-trip time delay between IoTs, end-users, and the cloud. Therefore, we propose a novel hybrid fuzzy-based RL algorithm employing NN evolution strategies and an analytical model to minimize the high latency. The present study aims to minimize the latency, network usage, and RAM consumption between healthcare IoTs, end-users, and cloud. The proposed algorithm and analytical model meet the QoS requirements for healthcare IoTs.

## Materials and methods

Healthcare ECG sensor data were obtained from the online (web source) UCI machine learning repository, which is a center for machine learning and intelligent system [38–42]. In our simulation, the ECG sensor data comprise 14 attributes and 303 instances. However, the original heart disease dataset from the UCI repository comprises 76 attributes. The dataset used in our simulation is a uniformly sampled data. The proposed algorithm was tested on an ECG dataset that includes data from a patient suffering from heart disease owing to high blood pressure, high sugar level, and high cholesterol. The data were recorded continuously. A total of 303 patients’ ECG strips/records were obtained from two leads (one channel). The attribute characteristics of the health dataset are categorical, integer-based, and real. See Table 5 for the data dictionary.

**Table 5:** Data dictionary for the dataset used in our simulation [38].

Variable	Definition
1. Age	Patient age in years.
2. cp	Chest Pain (Value 1: Angina, Value 2: Atypical form of angina, Value 3: Non-angina, Value 4: No symptoms of angina
3. restecg	ECG sensor resting value (Value 0: Normal, Value 1: Abnormal (ST-T wave), Value 2: Definite Ventricular
4. thalrest	Heart Rate Values at rest.
5. trestbps	Resting blood pressure value in mmHg.
6. chol	Cholesterol Value in mg/dl
7. fbs	Fasting Blood Sugar Value > 120mg/dl (True: 1 and False: 0)
8. num	Diagnosis of heart disease (Value 0: < 50% diameter narrowing, Value 1: > 50% diameter narrowing)
9. ca	Total number of major vessels.
10. thalach	Maximum heart rate recorded value.
11. sex	(1 = Male) or (0 = Female)
12. exang	Exercise induced angina.
13. oldpeak	ST Depression induced from exercise with respect to rest.
14. slope	Slope related to peak of exercise (Value 1, Value 2 and Value 3)

### System overview

The Q-learning Markov decision process (MDP) algorithm was used under the constraint to achieve the minimum computation latency, communication latency, and network latency by allocating data packets to different processors of virtual machines. Q-learning MDP is a mathematical framework for modeling decision-making and observations by collecting feedback from past experience in a dynamic environment [43]. The proposed approach requires a Q-learning MDP to account for the dynamic behavior of the IoT-fog-cloud system [23, 43]. The IoT-fog-cloud system was unable to predict the transition probabilities and rewards because of dynamically changing incoming data packet requests at fog nodes. A decision-making process has been established using Q-learning MDP to mitigate the problem of different data packet demands from different users at different time intervals and computational capacities of fog nodes. The Q-learning algorithm solves the MDP with unknown larger rewards and transition functions by exploring and exploiting the different states of the system [23]. Furthermore, it maximizes the total reward for the IoT-fog-cloud system using quality action.

A fuzzy-based RL algorithm was used to monitor PHD in real time. The characteristics of healthcare IoT requires RL to trace the patient background health state in minimum time [21]. The selection of data packets for computation in different fog nodes was performed using RL and a NN [44]. This further balanced the load among the nodes to transfer the data to end-users in minimal time. RL supports the optimum use of available resources by allowing the allocation of distinct data packets to processors without violating QoS barriers for delayed critical workloads [4, 45]. It was designed to obtain feedback from the patient’s previous health record, where the decision for constructing rules was processed by the FIS [23, 37, 44, 46]. The PHD were classified into low, normal, and high risk using fuzzy membership functions and fuzzy rules defined in FIS [47, 48]. Next, RL identified the best outcome of the action to maximize its total reward and the performance of the algorithm in a given time. The proposed algorithm was observed to reduce high latency.

**Fig 2:**
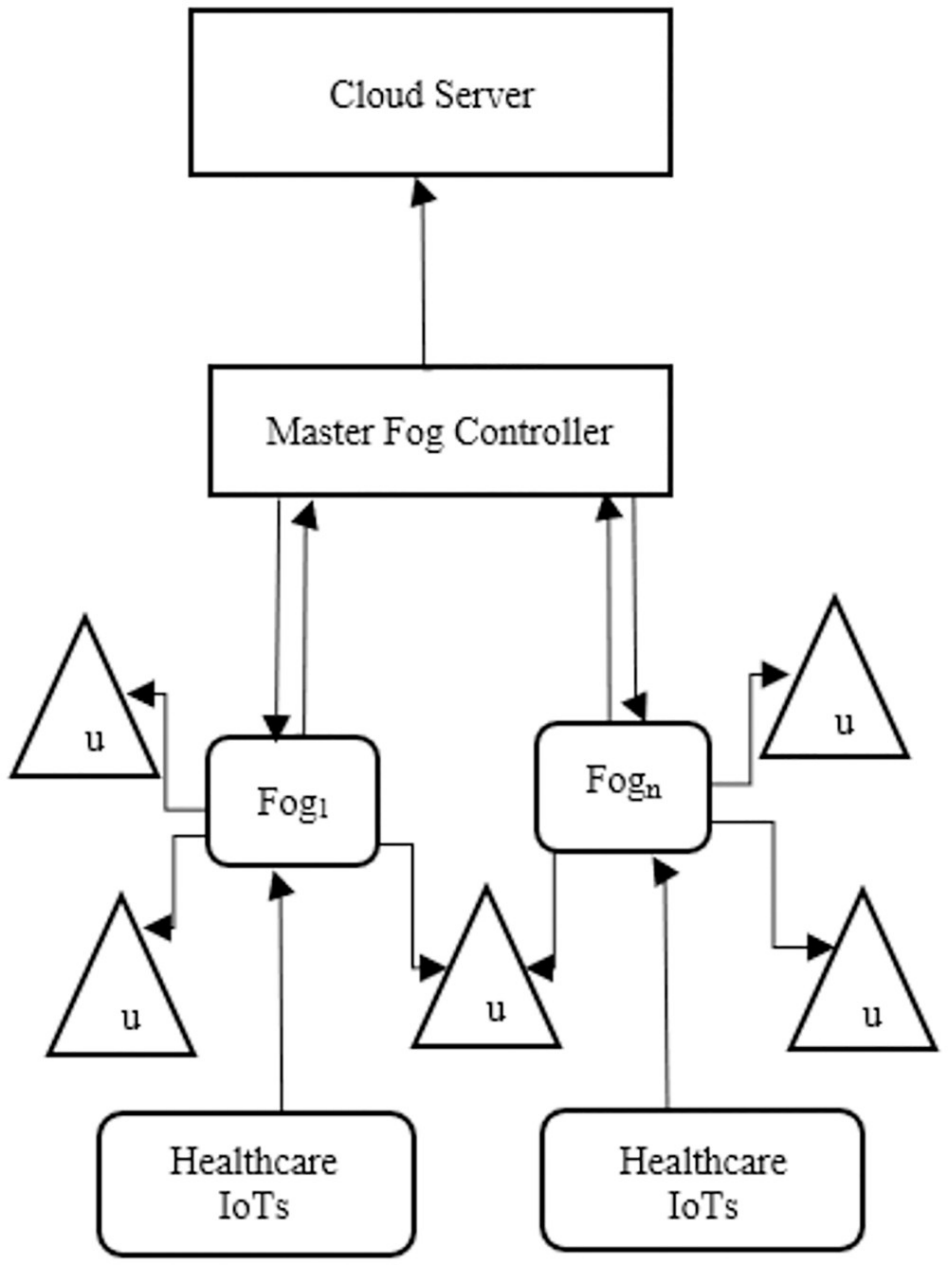
The healthcare IoT data transmission model consists of fog nodes, master fog controller, end-users (u) and cloud server.

Fig 2 shows the healthcare IoT data transmission model, in which the IoT sends a data packet to fog nodes. Subsequently, the fog nodes directly send the data packet to end-users. A master fog controller controls the fog node’s data transmission and selection and communicates further to a cloud server.

The process in the proposed model allows fog nodes to select appropriate data transmitted from IoT devices. Next, PHD is computed and transferred to other fog nodes and end-users in real time. This process is designed to reduce the high latency, i.e., reduces the total latency between IoTs and end-users. Distributed intelligent decision-making is required for the distribution of data packets to other fog nodes for computation. This distribution of data packets is associated with the requirement of data in minimal required time by end-nodes. The decision of data packet distribution considers the communication delay, prolonged computation delay, and network delay. A delay occurs between nodes owing to the transmission of a large number of data packets over a network. Crucial decisions include (i) which data packets for computation should be allocated to fog nodes to be sent in real time, (ii) How many data packets should be uploaded and migrated, and (iii) Scaling of data packets to fog nodes. It is noteworthy that the existing schemes emphasize primarily load offset and coordinated migration in a fog environment [19]. Studies to minimize the total latency among IoTs, end-users, and cloud servers using intelligent FC based on a hybrid machine learning approach have not been conducted. Generally, most previous studies do not convey the practical aspects of fog networks [19, 49]. The proposed new algorithm prepares the data allocation issue in the form of an MDP and is accountable to the change in system from the context of fog nodes. This problem allows fog nodes to move their data packets after allocation and computation to other fog nodes, which further transfer them to end-users. The proposed fuzzy-based Q-learning model in fog networks differs from existing approaches based on two main aspects. First, the dynamic environment is based on the end-users’ request for time-sensitive data packets from different distributed fog nodes. The interhop gap among neighboring fog nodes results in a change in the decision-making process for actions that are then selected to minimize service latency. Second, the network traffic control in a fog network refers to efficiency in data packet distribution to a fraction of fog nodes such that users can more easily access data in real time. It is a type of dynamic data packet allocation schema where the fog node allocates only data that are time-sensitive or requested by users. In our proposed method, fog nodes are defined as a server that can perform communication while exhibiting processing and computing capacity.

### Analytical model

#### System model

**Fig 3:**
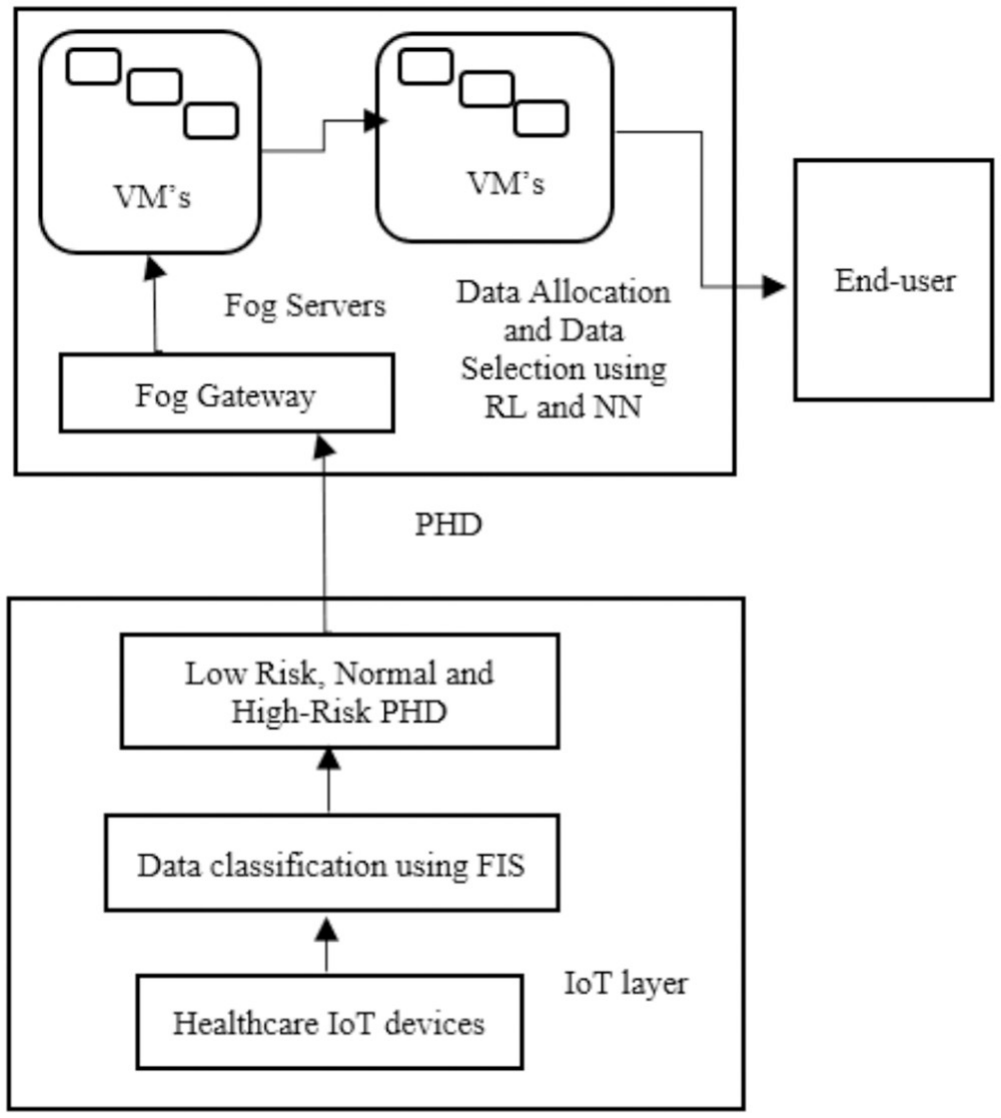
The healthcare IoT system model consists of healthcare IoT devices, classified PHD, fog gateways, fog servers, and virtual machines (VM’s).

Fig 3 shows the healthcare IoT system model for the FC environment. The data transmitted from healthcare IoT devices are classified into low risk, normal, and high risk by applying a FIS classification process. PHD are allocated through RL in various virtual machines in fog servers. The time-sensitive data are selected using an NN and sent to end-users within the minimum required time. In virtualization, fog nodes are used in the distribution and allocation of data packets among other nodes and end-users. End nodes are linked to fog nodes, where information retrieval can be sent.

A master fog node controller that contains the topology information of data packet allocation and distribution is used. Nodes are connected through a network topology and all the nodes are further connected to the master fog node. This study investigates a progressive data packet allocation approach using fog computation in the machine learning environment. The nodes can transfer data packets to other nodes to minimize latency and network traffic. Here, the CPU comprises data packets in a queue, which can be used as a good traffic index on nodes and affects the average response time. Each fog node can gather information, make decisions, serve the end nodes, and provide data on assembling traffic information and queue position. The master fog creates a network table by considering the information distributed from other nodes. The master fog node sends requests to determine whether the current node should move the required data. If so, data will be moved to the neighboring node, where selection is performed based on time and requisite data. The main objective of this study is to select time-sensitive data while reducing latency and network traffic.

### Problem formulation

To accomplish the requisite execution, the suggested problem of latency minimization in healthcare IoTs and cloud was developed as an MDP, for which an algorithm with a performance guarantee [22, 23, 27] is proposed. The MDP comprises a fog node in the form of a selection agent that regularly inspects the instant conditions of the controlled system, selects between those who have permission in the state (*a* ∈ *A*_*i*_(*s*)), and then detects the progression in a different state *s*′ and reward ***r***, which will transform its subsequent selections. In the MDP, the agent selects an action as the work of states. Therefore, the new state and reward transition probability distribution will be observed. In our system, the master fog node selects an action as a function of the current state and considers the reward shown in the following states and all nodes. Our MDP is characterized by a 4-tuple 〈*Si*, *A*_*i*_, *P*_*i*_, *R*_*i*_〉, where $S_{i}=\{s=\left(n_{l},d_{l}^{p},Q\right)\}$ is the state space, *n*_*l*_ ∈ *N*(1 ≤ *n*_*l*_ ≤ *N*) is the node that contains the data packets for allocation as requested by the end-users, $d_{l}^{p}\in N\left(1\leq d_{l}^{p}\leq D_{{\text{max}}_{L}}^{P}\right)$ is the number of data packets to be allocated per unit time, *Q* = {*Q*_1_, − − − − −, *Q*_*N*_} | *Q*_*i*_ ∈{0, 1, − − − − −, *Q*_*i*,max_}} is the number of data packets and currently remaining in the queue of the fog node. Additionally,$A_{i}=\left\{a=\left(n_{f},d_{f}^{p}\right)\right\}$ is the action space, where *n*_*f*_ ∈ *N*(1 ≤ *n*_*f*_ ≤ *N*, *n*_*f*_ ≠ *n*_*l*_) is defined as the neighboring node within the fog network that is being allocated with data packets sent by node *n*_*l*_.

$d_{f}^{p}\in N\left(1\leq d_{f}^{p}\leq D_{{\text{max}}_{L}}^{P}\right)$ is the number of data packets sent to *n*_*f*_, which is a neighboring fog node.

Let *A*_*i*_(*s*) ∈ *A*_*i*_ be a group of actions that can be performed on state *s*. *A*_*i*_(*s*) is defined such that node *n*_*l*_ can only move the data packet to the second node and to the user with the same or a smaller number of data packets currently required. Based on the action *a*, the total count of data packets to be locally processed $\left(d_{f}^{l}\right)$ is decided with respect to the accessible queue space of node *n*_*l*_.

*P*_*i*_: *S*_*i*_ × *A*_*i*_ × *S*_*i*_ →[0, 1] is the transition probability distribution *P*_*i*_(*s*′, *a*) of a new state *s*′ from a given state *s* when an action *a* is performed.

*R*_*i*_: *S*_*i*_ × *A*_*i*_ →*R*_*i*_ is the reward when the system is in state *s* and action *a* is performed. The essential objective of the system is to perform a peerless data packet allocation operation on each node to maximize the utility while reducing latency and data packet allocation probability. Therefore, the determined system characterizes the instant reward function *R*_*i*_(*s*, *a*) given action *a* at state *s* as follows:

$$R_{i}\left(s,a\right)=U_{i}\left(s,a\right)-\left(L_{i}^{FOG}\left(s,a\right)+O\left(s,a\right)\right),$$ (1)

where *U*_*i*_(*s*, *a*), $L_{i}^{FOG}\left(s,a\right)$ and *O*(*s*, *a*) represent the instant utility, instant latency, and data packet allocation probability function, in combination, respectively.

The instant utility is computed as

$$U_{i}\left(s,a\right)=r_{iu}\text{log}\left(1+d_{f}^{l}+d_{f}^{p}\right),$$ (2)

where *r*_*iu*_ is the reward utility.

$$L_{i}^{FOG}\left(s,a\right)=\left(\chi_{l}.C_{L}^{FOG}+C_{PL}^{FOG}+N_{L}^{FOG}\right)/(d_{f}^{l}+d_{f}^{p}),$$ (3)

where *χ*_*l*_ is the latency weight. Here, $C_{L}^{FOG}$ is the communication latency, $C_{PL}^{FOG}$ the computation latency, and $N_{L}^{FOG}$ the network latency.

#### Communication latency

The round-trip times required by a data packet from an end-user node (wearable IoT device) to a fog node and from a fog node back to an end-user node (wearable IoT device) are determined, as follows:

$$C_{L}^{FOG}=C_{L}^{FOG}\left(\text{Re}quest\right)+C_{L}^{FOG}\left(\text{Re}sponse\right)$$

$$C_{L}^{FOG}\left(\text{Re}quest\right)=\frac{D_{P}^{S}}{v_{n_{e},n_{f}}}\cdot d_{l}^{p}$$

and

$$C_{L}^{FOG}\left(\text{Re}sponse\right)=\frac{D_{P}^{S}}{v_{n_{f},n_{e}}}\cdot d_{f}^{p}$$

$$C_{L}^{FOG}=\frac{D_{P}^{S}}{v_{n_{e},n_{f}}}\cdot d_{l}^{p}+\frac{D_{P}^{S}}{v_{n_{f},n_{e}}}\cdot d_{f}^{p},$$

where,

$$v_{n_{e},n_{f}}=B_{e_{w}}.\text{log}\left(1+\frac{g_{n_{e},n_{f}}.P_{t_{x}n_{e}}}{B_{e_{w}}.N_{0}^{e}}\right)$$

and

$$v_{n_{f},n_{e}}=B_{f_{w}}.\text{log}\left(1+\frac{g_{n_{f},n_{e}}.P_{t_{x}n_{f}}}{B_{f_{w}}.N_{0}^{f}}\right)$$

$C_{L}^{FOG}$ between the end-user node *n*_*e*_ and neighboring fog node *n*_*f*_ is determined, as

$$C_{L}^{FOG}=D_{P}^{S}\cdot \left(\frac{d_{{}_{l}}^{p}}{v_{n_{e},nf}}+\frac{d_{f}^{p}}{v_{n_{f},n_{e}}}\right),$$ (4)

$C_{L}^{FOG}$ between fog nodes *n*_*l*_ and *n*_*f*_ is expressed as

$$C_{L}^{FOG}=D_{P}^{S}\cdot \left(\frac{d_{f}^{l}}{v_{n_{l},n_{f}}}+\frac{d_{f}^{p}}{v_{n_{f},n_{l}}}\right),$$ (5)

where,

$$v_{{}_{n_{l},n_{f}}}=B_{l_{w}}.\text{log}\left(1+\frac{g_{n_{l},n_{f}}.P_{t_{x}n_{l}}}{B_{l_{w}}.N_{0}{}^{l}}\right)$$

and

$$v_{n_{f},n_{l}}=B_{f_{w}}.\text{log}\left(1+\frac{g_{n_{f},n_{l}}.P_{t_{x}n_{f}}}{B_{f_{w}}.N_{0}^{f}}\right)$$

$C_{L}^{FOG}$ between node *n*_*e*_ and node *n*_*l*_ is determined as

$$C_{L}^{FOG}=D_{P}^{S}\cdot \left(\frac{d_{l}^{p}}{v_{n_{e},n_{l}}}+\frac{d_{f}^{l}}{v_{n_{l},n_{e}}}\right),$$ (6)

where,

$$v_{{}_{n_{l},n_{e}}}=B_{l_{w}}.\text{log}\left(1+\frac{g_{n_{l},n_{e}}.P_{t_{x}n_{l}}}{B_{l_{w}}.N_{0}{}^{l}}\right)$$

and

$$v_{{}_{n_{e},n_{l}}}=B_{e_{w}}.\text{log}\left(1+\frac{g_{n_{e},n_{l}}.P_{t_{x}n_{e}}}{B_{e_{w}}.N_{0}{}^{e}}\right)$$

Here, $d_{l}^{p}$ is the number of data packets sent by *n*_*e*_ to *n*_*f*_ and *n*_*e*_, $d_{f}^{p}$ the number of data packets sent by *n*_*f*_ to *n*_*e*_ and *n*_*l*_, $d_{f}^{l}$ the number of data packets sent by *n*_*l*_ to *n*_*e*_ and *n*_*f*_, $D_{P}^{S}$ the size of data packets, $v_{n_{e},n_{f}}$ the IoT device transmission service rate from *n*_*e*_ to *n*_*f*_, $v_{{}_{n_{f},n_{e}}}$ the fog transmission service rate from *n*_*f*_ to *n*_*e*_, $v_{n_{e},n_{l}}$ the transmission service rate from *n*_*e*_ to *n*_*l*_, $v_{{}_{n_{l},n_{e}}}$ the fog transmission service rate from *n*_*l*_ to *n*_*e*_, $v_{n_{l},n_{f}}$ the transmission service rate from *n*_*l*_ to *n*_*f*_, $v_{n_{f},n_{l}}$ the transmission service rate from *n*_*f*_ to *n*_*l*_, $B_{e_{w}}$,$B_{f_{w}}$, and $B_{l_{w}}$ the bandwidths per a node *n*_*e*_, *n*_*f*_ and *n*_*l*_, $g_{n_{e},n_{f}}≜\beta_{1}d_{n_{e},n_{f}}{}^{-\beta_{2}}$, $g_{n_{f},n_{e}}≜\beta_{3}d_{{}_{n_{f},n_{e}}}{}^{-\beta_{4}}$, $g_{n_{l},n_{e}}≜\beta_{5}d_{n_{l},n_{e}}{}^{-\beta_{6}}$, $g_{n_{e},n_{l}}≜\beta_{7}d_{n_{e},n_{l}}{}^{-\beta_{8}}$, $g_{n_{l},n_{f}}≜\beta_{9}d_{n_{l},n_{f}}{}^{-\beta_{10}}$, and $g_{n_{f},n_{l}}≜\beta_{11}d_{n_{f},n_{l}}{}^{-\beta_{12}}$ the channel gains for $v_{n_{e},n_{f}}$, $v_{{}_{n_{f},n_{e}}}$, $v_{{}_{n_{l},n_{e}}}$, $v_{n_{e},n_{l}}$, $v_{n_{l},n_{f}}$ and $v_{n_{f},n_{l}}$, $d_{n_{e},n_{f}}$ the distance between nodes *n*_*e*_ and *n*_*f*_, $d_{{}_{n_{l},n_{e}}}$ the distance between nodes *n*_*l*_ and *n*_*e*_, $d_{{}_{n_{l},n_{f}}}$ the distance between nodes *n*_*l*_ and *n*_*f*_, $P_{t_{x}n_{e}}$, $P_{t_{x}n_{f}}$, and $P_{t_{x}n_{l}}$ the transmission powers of nodes *n*_*e*_, *n*_*f*_ and *n*_*l*_, $N_{0}^{e}$ the noise power density for transmission service rate from *n*_*e*_ to *n*_*f*_ and *n*_*l*_, $N_{0}^{f}$ the noise power density for transmission service rate from *n*_*f*_ to *n*_*e*_ and *n*_*l*_, $N_{0}^{l}$ the noise power density for transmission service rate from *n*_*l*_ to *n*_*e*_ and *n*_*f*_, *β*_1_, *β*_3_, *β*_5_, *β*_7_, *β*_9_ and *β*_11_ denotes the path loss constant, and *β*_2_, *β*_4_, *β*_6_, *β*_8_, *β*_10_ and *β*_12_ denotes the path loss exponent, respectively.

#### Network latency

By assuming the same latency for every hop delay, the network latency depends on the total packets sent from end-user node *n*_*e*_ to fog node *n*_*l*_, *n*_*l*_ to fog node *n*_*f*_, and from *n*_*f*_ to *n*_*e*_; the network latency is expressed as

$$N_{L}^{FOG}=\frac{l_{n}H_{C}n_{l}+l_{n}H_{C}n_{f}+l_{n}H_{C}n_{e}}{T_{P}},$$

$$N_{L}^{FOG}=\frac{l_{n}H_{C}\cdot (n_{l}+n_{f}+n_{e})}{T_{P}},$$ (7)

here

$$T_{P}=d_{l}^{p}+d_{f}^{l}+d_{f}^{p}$$

where *H*_*C*_ is the number of hop counts, *T*_*P*_ the total data packets sent, and *l*_*n*_ the unit hop delay.

#### Computation latency

By assuming a query system and neglecting packet loss, with the data packet arrival rate and service rate for the fog node, the computation latency(waiting time and service time) can be expressed as

$$C_{PL}^{FOG}=\frac{N_{I}\cdot CPU_{I}\cdot d_{f}^{l}}{c_{s}^{l}}+\frac{N_{I}\cdot CPU_{I}\cdot d_{f}^{p}}{c_{s}^{f}}+\frac{1}{v_{{}_{n_{l},n_{e}}}-\lambda_{e}}+\frac{1}{v_{{}_{n_{l},n_{f}}}-\lambda_{f}}+\frac{1}{v_{{}_{n_{f},n_{l}}}-\lambda_{l}}+\frac{1}{v_{{}_{n_{f},n_{e}}}-\lambda_{e\prime }},$$ (8)

where *N*_*I*_ is the total count of instructions per data packet, *CPU*_*I*_ the CPU cycle per instruction, *λ*_*e*_, *λ*_*l*_, *λ*_*e*′_ and *λ*_*f*_ are the data packet arrival rates at nodes *n*_*l*_ and *n*_*f*_, $c_{s}^{l}$ and $c_{s}^{f}$ the CPU speeds of nodes *n*_*l*_ and *n*_*f*_.

The data packet allocation probability O(*s*, *a*) is calculated as

$$O\left(s,a\right)=\frac{\chi_{i}\left(d_{f}^{l}\cdot P_{allocation,l}+d_{f}^{p}.P_{allocation,f}\right)}{d_{f}^{l}+d_{f}^{p}},$$ (9)

$$P_{{}_{allocation,i}}=\frac{\text{max}\left(0,\lambda_{i}-\left(Q_{i,\text{max}}-Q_{i}{}^{\prime }\right)\right)}{\lambda_{i}},$$ (10)

$$Q_{i}{}^{\prime }=\text{min}(\text{max}(0,Q_{i}-v_{i})+d_{f_{i}}^{l},Q_{i,\text{max}}),$$ (11)

*χ*_*i*_ is the data packet allocation weight, *v*_*i*_ the service rate of a node *n*_*i*_, $d_{f_{i}}^{l}$ the total count of data packets to be locally processed at node *n*_*i*_, and *λ*_*i*_ is the data packet arrival rate at node *n*_*i*_. $Q_{i}{}^{\prime }$ represents the next queue state, i.e., remaining data packets of a node *n*_*i*_ in state *s* when an action *a* is performed. The total latency is then expressed as

$$T_{L}=C_{L}^{FOG}+N_{L}^{FOG}+C_{PL}^{FOG}$$ (12)

$$\begin{array}{l} T_{L}=D_{P}^{S}\cdot \left(\frac{d_{l}^{p}}{v_{n_{e},n_{l}}}+\frac{d_{f}^{l}}{v_{n_{l},n_{e}}}\right)+D_{P}^{S}\cdot \left(\frac{d_{f}^{l}}{v_{n_{l},n_{f}}}+\frac{d_{f}^{p}}{v_{n_{f},n_{l}}}\right)+D_{P}^{S}\cdot \left(\frac{d_{l}^{p}}{v_{n_{e},n_{f}}}+\frac{d_{f}^{p}}{v_{n_{f},n_{e}}}\right)+\frac{l_{n}H_{C}\cdot \left(n_{l}+n_{f}+n_{e}\right)}{T_{P}}+\\ \frac{N_{I}\cdot CPU_{I}\cdot d_{f}^{l}}{c_{s}^{l}}+\frac{N_{I}\cdot CPU_{I}\cdot d_{f}^{p}}{c_{s}^{f}}+\frac{1}{v_{{}_{n_{l},n_{e}}}-\lambda_{e}}+\frac{1}{v_{{}_{n_{l},n_{f}}}-\lambda_{f}}+\frac{1}{v_{{}_{n_{f},n_{l}}}-\lambda_{l}}+\frac{1}{v_{{}_{n_{f},n_{e}}}-\lambda_{e\prime }} \end{array}$$

The data traffic rate is to be sent through a one-hop transmission path from fog nodes *n*_*l*_ and *n*_*f*_ to an end-user node *n*_*e*_. It is important to certify the QoS (latency requirement) for end-users. Owing to large data transmission and high data traffic, end-user experience several delays including computation latency (delay in queues on nodes), communication latency, and network latency. The purpose of the proposed method is to reduce latency, with the transition probability *P*_*i*_ and reward *R*_*i*_ determined before the execution of the system. In each state, the optimum action is defined as a series that yields the maximum long-term reward, which is the disclosure sum of the expected recent rewards of all future decisions regarding the state-action that begins with the present state. In the future, the instant reward obtained in *k*′more time steps is worth $\gamma_{i}{}^{k^{\prime }-1}$ times, where *γ*_*i*_ is labeled as a discount factor (0 < *γ*_*i*_ < 1). The highest value function is determined, which satisfies the Bellman optimality equation:

$$\begin{array}{rr} \nu^{*}\left(s\right) & =\underset{a}{\text{max}}\;\text{E}\left(R_{i_{t+1}}+\gamma_{i}\nu^{*}(S_{i_{t+1}})|S_{i_{t}}=s,A_{i_{t}}=a\right)\\ & =\underset{a}{\text{max}}\sum_{s\prime ,r}p_{i}\left(s\prime ,r|s,a\right)\left[r+\gamma_{i}\nu^{*}\left(s\prime \right)\right] \end{array}$$ (13)

### Mathematical framework for latency minimization

In maximum events, the system cannot accurately predict probability *P*_*i*_ and reward *R*_*i*_ because the system can cause variations in those parameters. To discourse this limitation, RL is suggested. In RL, the loss of confidential data is solved by observing background details [44]. The canonical decision-making algorithm has limited functionalities in RL owing to its hypothesis of an ideal model and its considerable estimation value [21]. Q-learning is a canonical model-free algorithm [44] that is frequently applied to the acquisition of the highest state-action method for any MDP. For the proposed system, the learning master fog node acts as a controller that continuously detects the present state *s* with an action *a*, followed by a transition. Subsequently, it detects the different state *s*′ and the reward *r*. With these detections, it manages and renews its projections of the Q-function such that the following is obtained:

$$Q\left(s,a\right)\leftarrow \left(1-\alpha_{i}\right)Q\left(s,a\right)+\alpha_{i}\left[R_{i}\left(s,a\right)+\gamma_{i}\underset{a\prime \in A_{i_{s\prime }}}{\text{max}Q\left(s\prime ,a\prime \right)}\right],$$ (14)

where *α*_*i*_ is the learning rate (0 < *α*_*i*_ < 1); here, *α*_*i*_ balances the weight of old estimation with the weight of new estimation and observation. Eq 14 is a classic MDP in which Q stands for the quality of action *a* on state *s* and *Q*(*s*′, *a*′) is the Q function for transition state *s*′ and action *a*′. The equation solves the issue of transition states and rewards for the healthcare IoT-fog-cloud system. The main fog node in the system acts as a controller to monitor the current state and action. The fog node further collects the information on new states *s*′ and rewards *r*. Once the transition is completed, the Q-function is updated as shown in Eq 14. This equation overcomes the problem of change in the transition probability function rewards using the classic RL technique, namely, Q-learning MDP. The intelligible action choice rate is to collect the single action with the maximal approximate rate, i.e., greedy selection (*a*_*t*_ ≐ arg max_*a*_ *Q*_*t*_(*a*)). Thus, the greedy action choice rate consistently obtains the present knowledge to exaggerate the current reward, which is an essential aspect of the Q-learning ∊-greedy algorithm [23]. The algorithm acts greedy for a greater number of terms, but includes a limited possibility that ∈ haphazardly chooses against the complete accessible actions amidst the same number of probabilities. RL calls the greedy selection and ∈ probability of random selection as the greedy choice of exploitation and exploration approach [44, 45]. Exploitation is the appropriate action to exaggerate the requisite reward at a step, while exploration can generate the maximum overall long-term reward [4].

One application of the ∊-greedy algorithm is when the fraction of moves is incremented, the entire action is determined to be a converse immeasurable fraction of the total duration, thus certifying that *Q*(*s*, *a*) is the optimum value [21]. The estimated reward function, selected by the proposed approach, is calculated using Eq (1). After defining its three components, the next state *s*′ is obtained. Whereas the neighboring queue of the state is an arbitrary unit, the adjacent fog node has the function of sending data packets for allocation to other fog nodes. After the arrival of the data packet, the data size is determined at the fog node.

The demand for localized and location-based information services from patients/end-users is high. End-users are unable to retrieve time-critical localized data from cloud servers; thus, the FC approach is used [50, 51]. Depending upon the user requirement, fog nodes deploy the local computing facilities at the user end. Fog nodes deliver stored cloud data to mobile users with fast local connections [52]. Fog devices can be a hardware router, switches, IP video cameras, etc. A fog server can be a virtualized computing system and a lightweight cloud server.

A mathematical framework is presented to investigate the latency-delay tradeoff by process allocation in the FC environment. FC can provide a low latency response for time-sensitive applications. Low latency is required for the *i*-th service distribution and the completion phase. Communication latency depends on multiple channel factors (medium transmission capacity; connection between resources and interface). Computing latency is managed by the fog node itself. In our proposed method, RL is used for a long-term period; RL uses progressive strategies to allocate real-time data packets between fog servers to reduce the total latency. It has been observed that total latency includes communication, computing, and network latency. To adapt to different networking environments, we considered data packets in the form of a random and independent packet to be propagated over a communication channel between IoTs and fog servers. Let $\left(s_{i}^{d},c_{i}^{d},\tau_{i}^{d}\right)$ denote a three-dimensional characteristic vector of the *i*-th data packet, where $\langle s_{i}^{d},c_{i}^{d},\tau_{i}^{d}\rangle$ is the packet size, complexity, and latency limit of the data packet, in combination. Further, $f_{j}^{CPU}$ and $b_{j}^{s}$ indicate the frequency of the CPU in Hz and the current storage size of the *j*-th fog server, respectively. Consider that the *i*-th data packet ascribes to the *j*-th fog server; then the latency of the *i*-th data packet at the time of allocation to the fog servers is expressed by

$$l_{ij}=\frac{s_{i}^{d}c_{i}^{d}+b_{j}^{s}}{f_{j}^{CPU}}$$ (15)

Long-term optimization is achieved by selecting a fog server to assign the required data packet for allocation. Once data computation is complete at the fog server, the data are sent to the end-user, which further minimizes the total latency.

To express this problem mathematically, let *y*_*ij*_ denote the case when the *i*-th data packet is assigned to the *j*-th fog server. The latency minimization function at time slot *t* is characterized by

$$Ρ\left(t\right)≜\text{min}\sum_{i=1}^{\Omega \left(t\right)}\sum_{j=1}^{\Psi }y_{ij}l_{ij}$$ (16)

$$\sum_{i=1}^{\Omega \left(t\right)}y_{ij}=1,\forall j\in \Psi ,$$ (17)

$$l_{ij}\leq \tau_{i}^{d},\forall j\in \Psi$$ (18)

$$y_{ij}\in \left\{0,1\right\},\forall i\in \Omega \left(t\right),\forall j\in \Psi$$ (19)

where Ω(*t*) and ψ are the sets of data packets and fog server, respectively. Therefore, the long-term latency reduction function (*f*_Δ_) is given by

$$\left(f_{\Delta }\right)\;\underset{t\to \infty }{\text{lim}}\frac{1}{t}\sum_{i=1}^{t}Ρ\left(i\right)$$ (20)

To fully process the uploaded data packet, the computation latency of the system becomes maximal between the distributed fog servers. The decision is made using the greedy method, which reduces the system latency when data packets are uploaded.

#### Fuzzy-based RL algorithm for real-time PHD transmission

The proposed algorithm is divided into two-sub algorithms: Algorithm 1 and 2.

Algorithm 1: Healthcare data is classified using an (FIS). Here, the tuples are created and merged in a fuzzy system that is used as input to the FIS. Fuzzy sets are created for the final values, followed by the FIS for PHD classification.

**Algorithm1** Patient Health Data (PHD) classification using FIS.

Algorithm Symbol Notations:

*f*_*G*_: Fog gateway for allocation of the data packet

*μ*_1_: Fuzzy Inference System membership function

*RTA*: Real-Time Analyzer

*P*_*id*_: Patient id

1: (Fuzzy system) the system is created with inputs and their member functions *μ*_1_

2: With the function, *μ*_1_(HeartRate1), *μ*_1_(ECG1), get the condition of health as *μ*_1_ (normal) or *μ*_1_(low risk) or *μ*_1_(high risk)

3: **IF** (Health Condition = *μ*_1_ (high risk))

THEN get geo-location

send

A notification to *f*_*G*_ using SPARK as *RTA*

4: **ELSE IF** (Health Condition = *μ*_1_(low risk)

THEN the *P*_*id*_ is sent to *f*_*G*_

5: END

In our proposed method, the training environment is a system comprising fog servers. In a model with RL, an action selection function exists (i.e., data packet selection and data packet allocation to fog server in real time); the function selects actions stationed on the state of the system.

Values are available to define and express the system state. These values are the (1) demand (complexity and size of data packets), (2) remaining data packets in fog storage, (3) time consumed from the last instant to upload the data packet to the present instant, and (4) series of requirements from the final data packet. When the data packet is uploaded for allocation to the fog server, we need to measure the time duration required by the server to complete the computation of the previous remaining data packets, e.g., the computation latency of the servers, and submit the value in the form of a [*K* × 1] vector. Subsequently, we determine the duration of time that the server will allocate if the arrival data packet is accredited. The value is saved in an additional vector of similar size [*K* × 1]. Combining the two abovementioned vectors, we obtain a [2*K* × 1] vector that exemplifies the state of the system near a given time. We quantify the computational latency, in microseconds, for fog servers to allocate data and send data packets. Data packets require minimal number of megacycles for its allocation; thereby, a deviation in expected latency occurs between the servers. Based on the latency in allocation, processing, and transmission of data packets, we create the state of the system in a vector form. The NN is selected as the action selection function in an RL model. The state is the input of the NN system. The size of the state is [2*K* × 1], the input layer of the NN contains 2*K* nodes *Z*^(*i*)^, *i* = {1, 2……*K*}, and the nodes are connected to every other node in the hidden layer. In the hidden layer, *M* nodes exist, denoted as *H*_*j*_, *j* = = {1, 2……*M*}. Consequently, a [*M* × *K*] network relationship exists between the input and the hidden layer. Every packet gains a weight, with the ability to store all the weights of the packets and a matrix *W*^*l*(1)^ is present. Weight $W_{i,j}^{l\left(1\right)}$ represents the relation between *Z*^(*i*)^ and *H*^*j*^ in row *i* and column *j*. The value of node *H*^*j*^ in the hidden layer is the gross summation of all the products of weights and inputs.

$$H^{j}=\sum_{i=1}^{2K}\left(W_{i,j}^{l\left(1\right)}\times Z^{\left(i\right)}\right)$$ (21)

The total count of nodes in the hidden layer can induce the training process. Hidden layer nodes are attached to the NN output layer, called the softmax layer [53]. The capacity of the output layer is [*K* × 1]. A matrix *W*(2) whose capacity is [*M* × *K*] stores the total weight of the network combination between the two layers. In the end layer, the node ${\hat{T}}^{\left(f\right)}$ value is calculated as

$${\hat{T}}^{\left(f\right)}=\sum_{j=1}^{M}\left(W_{j,f}^{\left(2\right)}\times Z^{\left(i\right)}\right)$$ (22)

After calculating the values of all the nodes, the time-sensitive PHD are transferred. Here, a fog server *fog*(*i*) is selected to transfer the time-sensitive PHD, where *i* = 1, 2 − − − − *K*. Its probability is given by

$$p_{fog\left(i\right)}=\frac{{\hat{T}}^{\left(i\right)}}{\sum_{f=1}^{K}{\hat{T}}^{\left(f\right)}}$$ (23)

To assign the uploaded data packet, the server with the highest probability is selected.

Eq 23 is derived using the softmax function [54]. The latter calculates the probability distribution for *k* real numbers and normalizes it into *k* probabilities, which are directly proportional to exponential functions of the input real numbers. Eq 23 is calculated using the softmax function formula, which is defined as $\sigma {\left(b\right)}_{j}=\frac{e^{b_{j}}}{\overset{k}{\underset{f=1}{\Sigma }}e^{b_{f}}}$ for *j* = 1, − − − −, *k*, and *b*(*b*_1_, − − − − − *b*_*k*_). The exponential function is applied to each element *b*_*j*_ of the input vector *b*. Next, normalization is performed to guarantee that the sum of the components of the output vector *σ*(*b*) is equal to 1. The softmax function is widely used in NN and RL. In RL, the softmax function is used to convert node values into probabilities [54].

**Fig 4:**
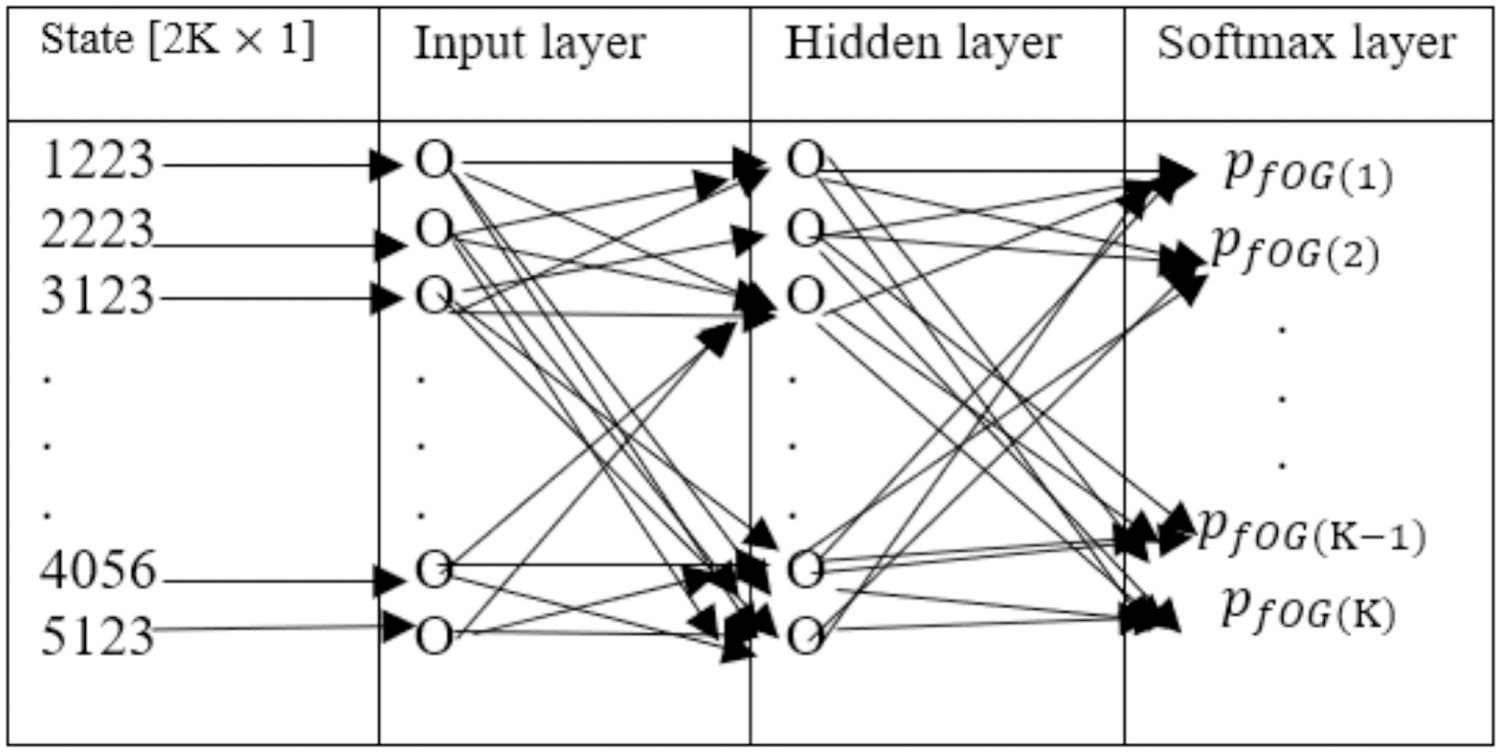
The NN states, an input layer, a hidden layer, and softmax layer.

Fig 4 shows the structures of the hidden and input layers in the NN of our proposed model. The NN is trained by restoring the weight matrices, e.g., *W*^(1)^ and *W*^(2)^, to exaggerate the response from the background details. In some RL-specific applications, backpropagation is performed to update the weight matrices [21, 55, 56]. However, this method is inefficient for the floating values of latency. Therefore, we selected a rival of the backpropagation method for NNs [55, 57, 58], which is called the NN evolution algorithm.

### Progression approach for the evolution

To optimize and train the machine learning model, a function is defined to assess the model performance in a problem established over an operational function. An RL model can determine the problems of data packet selection and allocation to fog servers in real time. Our aim is to select an action to reduce the long-term latency of the structural system, although RL can train the system to maximize the reward [59]. Here, the reward is inversely proportional to the system latency. After selecting an action *a*(*t*), the reward from the system is defined as

$$\text{Re}ward_{i}=\frac{1}{l(t)},$$ (24)

where

$$l(t)=l\left(t-1\right)+l_{ij}^{\left(t\right)},l(0)=0$$ (25)

Here, $l_{ij}^{\left(t\right)}$ is the latency produced by action *a*(*t*). Action *a*(*t*) is performed to assign the approaching *i*-th data packet to the *j*-th fog server. It is examined in a certain manner when *t* → ∞, *l*(*t*) → ∞ along with *Reward*_*i*_ → 0. Coordination by the latency minimization function (*f*_Δ_) in Eq (20), reduces the long-term latency of the system, which can be moderated by reducing the latency over the transition of *K* successive data packets for amplifying the equalization of rewards on *K* new actions. Hence, the reward is defined as

$$\text{Re}ward_{i}=\frac{1}{\sum_{f=t-K}^{t}l_{ij}\left(f\right)}$$ (26)

To upgrade the RL model for consecutive rewards, we restore the NN to increase the capacity of the model in selecting an action each for data packet selection and data packet allocation in the fog server. The most popular current algorithm in machine learning for updating an NN is backpropagation [55, 57, 60], which is feasible if the reward is either 0 or 1. Hence, backpropagation is no longer applicable considering our long-term reward. Neuroevolution (NE), i.e., neural network evolution, is used for training NNs [44, 55]. An NN is now assigned to each iteration; from the NN, a new generation is produced. This generation is a derivation of the NN [44, 57]. Children selection is based on a higher reward to renew the NN. To update the NN, evolution strategies are applied. It is now an accepted and recognized algorithm to apply to the NE method. [44, 55, 57].

Algorithm 2 illustrates the mechanism for data packet allocation and selection in real time. Algorithm 2 uses the greedy and NN approaches, where decisions are made by applying the greedy technique to minimize the latency of the schema at the time of data packet allocation. The NN is updated by evolution strategies in an RL environment [23, 44, 55]. For every repetition, *M* children of the NN are formed by the summation of Gaussian noise to each weight in the network. Every child in the NN performs a role each for data packet allocation and data packet selection in an RL model with *K* data packets and receive an average reward (*Mean*_*reward*_*i*_) over *K* actions.

$$W_{j,f}^{\left(i\right)}=W_{j,f}^{\left(i\right)}+\alpha_{i}\times \sum Gain_{i}{}^{\left(H\right)}\times W_{j,f}^{\left(i\right)\left(H\right)},H=\left[1,2-----,M\right],$$ (27)

where *H* and *α*_*i*_ are the total count of children and the learning rate, respectively.

**Algorithm 2** RL with the greedy method and evolution strategies.

1: **Input** learning rate (*α*_*i*_) and discount factor (*γ*_*i*_), exploration policy (∈), service rate (*v*_*i*_), data packet arrival rate (*λ*_*i*_), and distance vector (*D*_*v*_), Parent NN with a weight matrix *W*^(*i*)^, No. of children *M*. *i* = 1, 2

2: **Output** Data allocation table (*Q*)and Parent NN with maximum performance.

3: **Set** *Q*(*s*, *a*) = 0(∀*s* ∈ *S*_*i*_)(∀*a* ∈ *A*_*i*_(*a*)), *iter* ≔ 0, and *s* ≔ (1, 1{(*Q*_1_ − − − − − − − *Q*_*N*_)| *Q*_*i*_ = 0})

4: **While** (*iter* ≤ maximum iteration) **do**

5: Select *a* ∈ *A*_*i*_(*a*) applying ∊- greedy algorithm

6: Allocate the data packets conferring to action *a* and examine the next state *s*′ and reward *r*.

7: $Q\left(s,a\right)\leftarrow \left(1-\alpha_{i}\right)Q\left(s,a\right)+\alpha_{i}\left[R_{i}\left(s,a\right)+\gamma_{i}{\text{max}}_{a\prime \in A_{i_{s\prime }}}Q\left(s\prime ,a\prime \right)\right]$

8: *s* ← *s*′

9: *iter* ← *iter* + 1

10: **For** iteration in a predetermined range **do**

11: **For** *H* in range *M* **do**

12: *child*^(*H*)^ = Parent NN + random noise (*N*_0_), [*W*^(*i*)(*H*)^ = *W*^(*i*)^ + *noise*]

13: Evaluate

14: Calculate *Mean*_*reward*_*i*_

15: $Gain_{i}{}^{\left(H\right)}=\text{Re}ward_{i}{}^{\left(H\right)}-Mean\_reward_{i}$   *H* = 1, − − − − − −, *M*

16: Parent NN → Parent NN + $\alpha_{i}\times \sum_{H=1}^{M}Gain_{i}{}^{\left(H\right)}\times child^{\left(H\right)}W^{\left(i\right)}=\alpha_{i}\times \sum Gain_{i}{}^{\left(H\right)}\times W^{\left(i\right)\left(H\right)}$

17: Evaluate Parent NN

**18**: **END**

Subsequently, the maximal performing parent NN and data allocation table (*Q*) are obtained.

The method for this suggested Q-learning algorithm is conferred in algorithm 2. The algorithm explores the field that presents the optimum reward for the data packet selection and allocation problems in an RL model.

**Fig 5:**
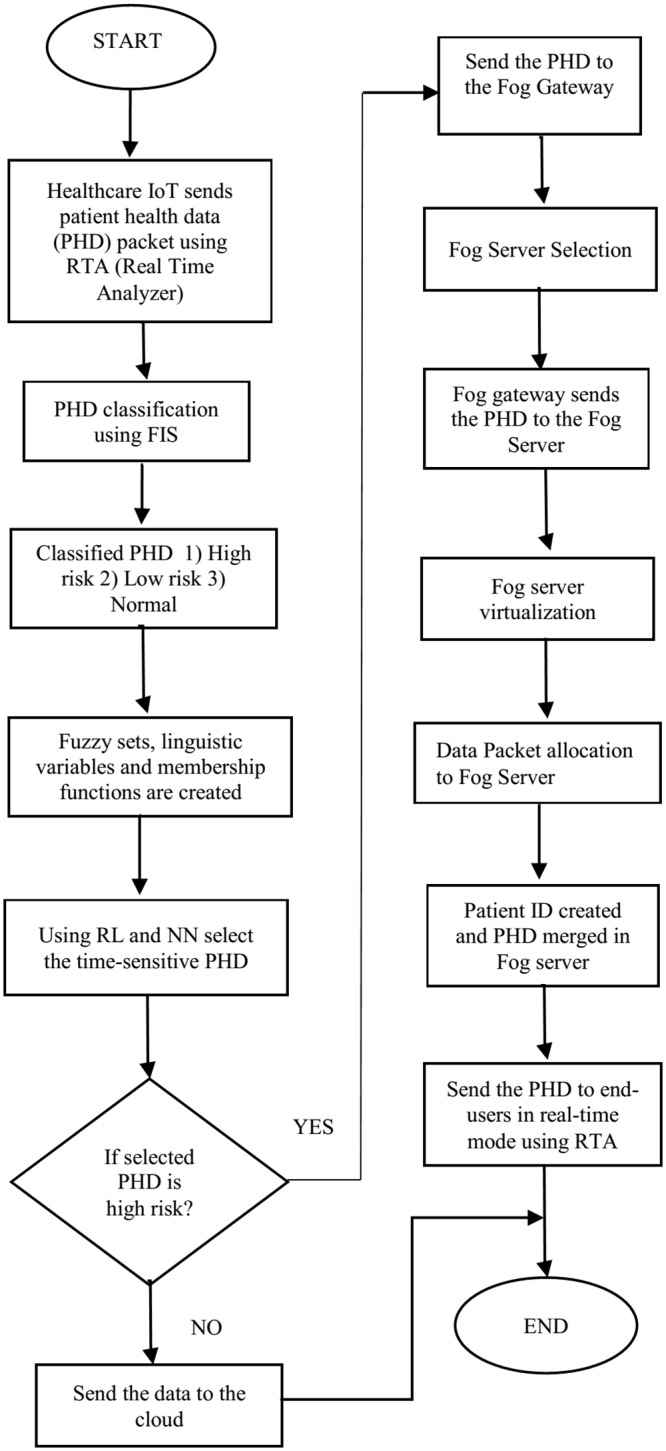
Algorithm flow chart for real-time data packet communication using RL, NN, and FIS in the FC environment.

Fig 5 illustrates the proposed algorithm flow for healthcare IoT data packet communication in real time using FC. Different procedural steps are shown for algorithms 1 and 2. The flow chart provides an insight into the proposed algorithm.

## Results and discussion

### Performance analysis and evaluation

In this section, the execution of the proposed analytical model with the proposed machine learning algorithm is evaluated and analyzed. A numerical test was conducted to verify the proposed hybrid machine learning algorithm and fog-based model. Predictive analysis using a support vector machine (SVM) was performed on PHD to examine the robustness of the performance measures. The key performance measures used to establish the validity and utility of our proposed algorithm are accuracy, sensitivity, specificity, positive predictive value (PPV), and negative predictive value (NPV).

#### Simulation overview

The performance of the FC model that incorporates the proposed algorithm is analyzed through simulation and experiments. The baseline for this simulation is minimum latency, minimum network usage, and minimum RAM consumption in cloud and FC environments. To simulate the FC-based analytical model, we used iFogSim [14] as an open-source software tool and the Python-based Spyder editor tool.

#### Performance analysis

**Fig 6:**
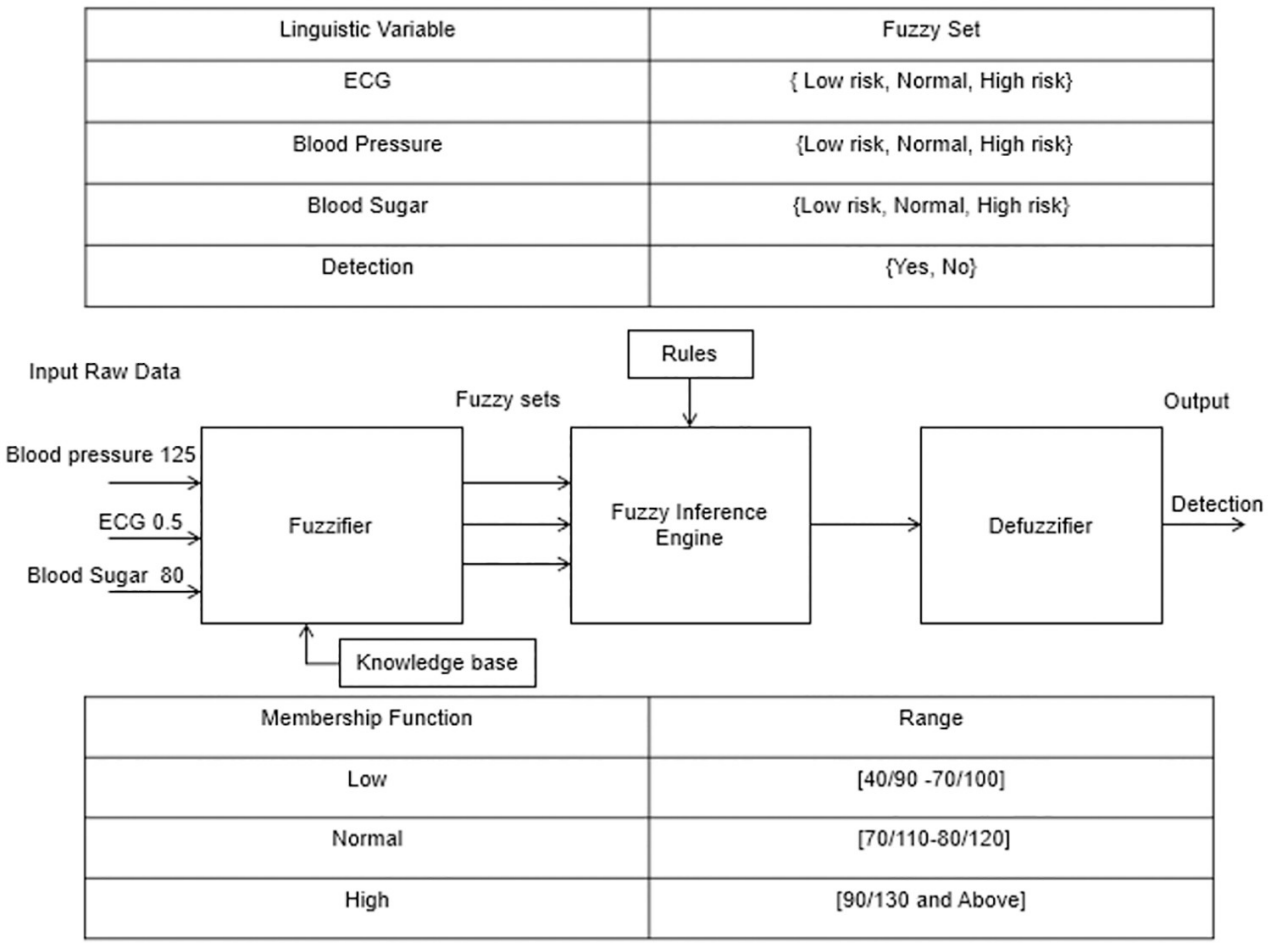
Schematic diagram of the FIS.

This subsection discusses the performances of the analytical model and the algorithms in terms of latency, network usage, and RAM consumption. The complete function of algorithm 1 is shown in Fig 6. A fuzzy control system is created using algorithm 1 in the Python editor tool. Algorithm 1 is used to classify ECG sensor data. We used the skfuzzy API to model the fuzzy system. Subsequently, to simulate algorithm 1, a control system is created. The control system defines the inputs, which is called the compute method. Once the simulation is completed, the results can be visualized. The data are classified using an FIS and a linear SVM. Using RL and NN evolutions strategies, algorithm 2 selects high-risk data (i.e., data with high ECG value) for data packet allocation and selection in various distributed fog servers.

Fig 6 shows the generated healthcare data. In the FIS, fuzzy sets are created with a fuzzy range of values. Next, we the output results are classified as low risk, normal, and high-risk health data based on the fuzzy rules and with member functions μ1 (HeartRate1) and μ1 (ECG1) to obtain the condition of health as μ1 (normal), μ1 (low risk), or μ1 (high-risk).

**Fig 7:**
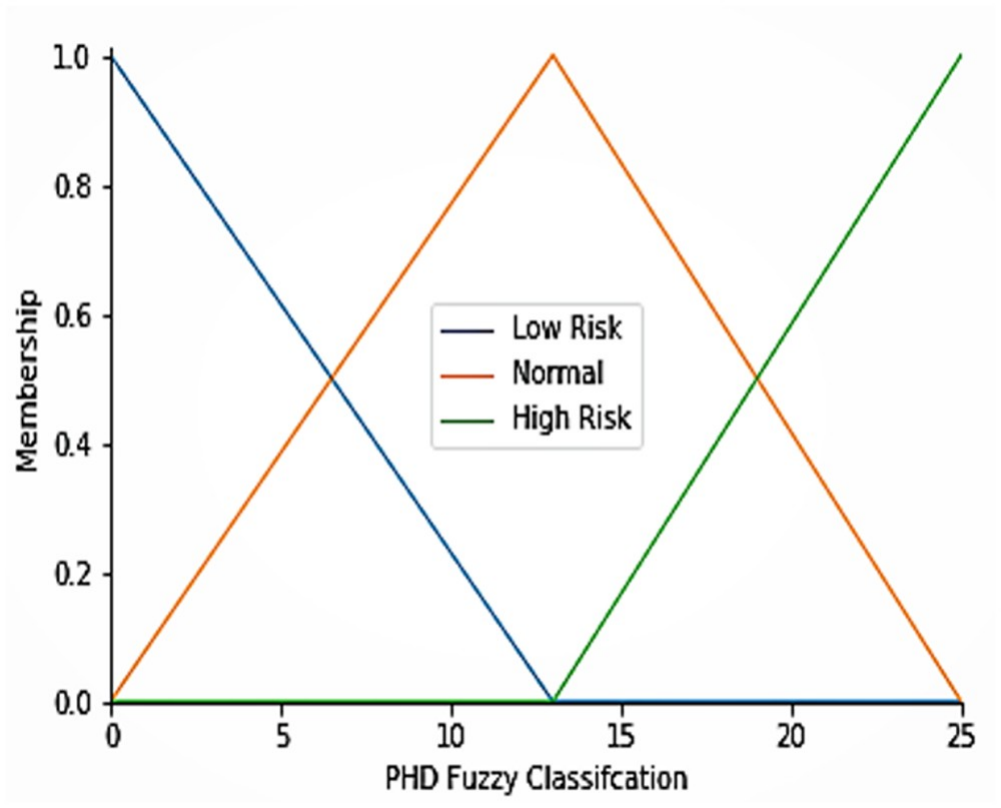
PHD classified as low risk, normal and high-risk using FIS and membership functions in the fuzzy logic system.

Fig 7 shows the low risk, normal, and high-risk healthcare ECG sensor data generated by algorithm 1 (as shown in Fig 6). The green line shows the high-risk ECG value with respect to the membership functions, whereas the red and blue lines show the normal and low-risk ECG values. Linguistic variables and fuzzy sets are created in the FIS. Membership functions are used as the ECG input and range. The next step is to regulate the extent to which input variables belong to the membership functions.

**Fig 8:**
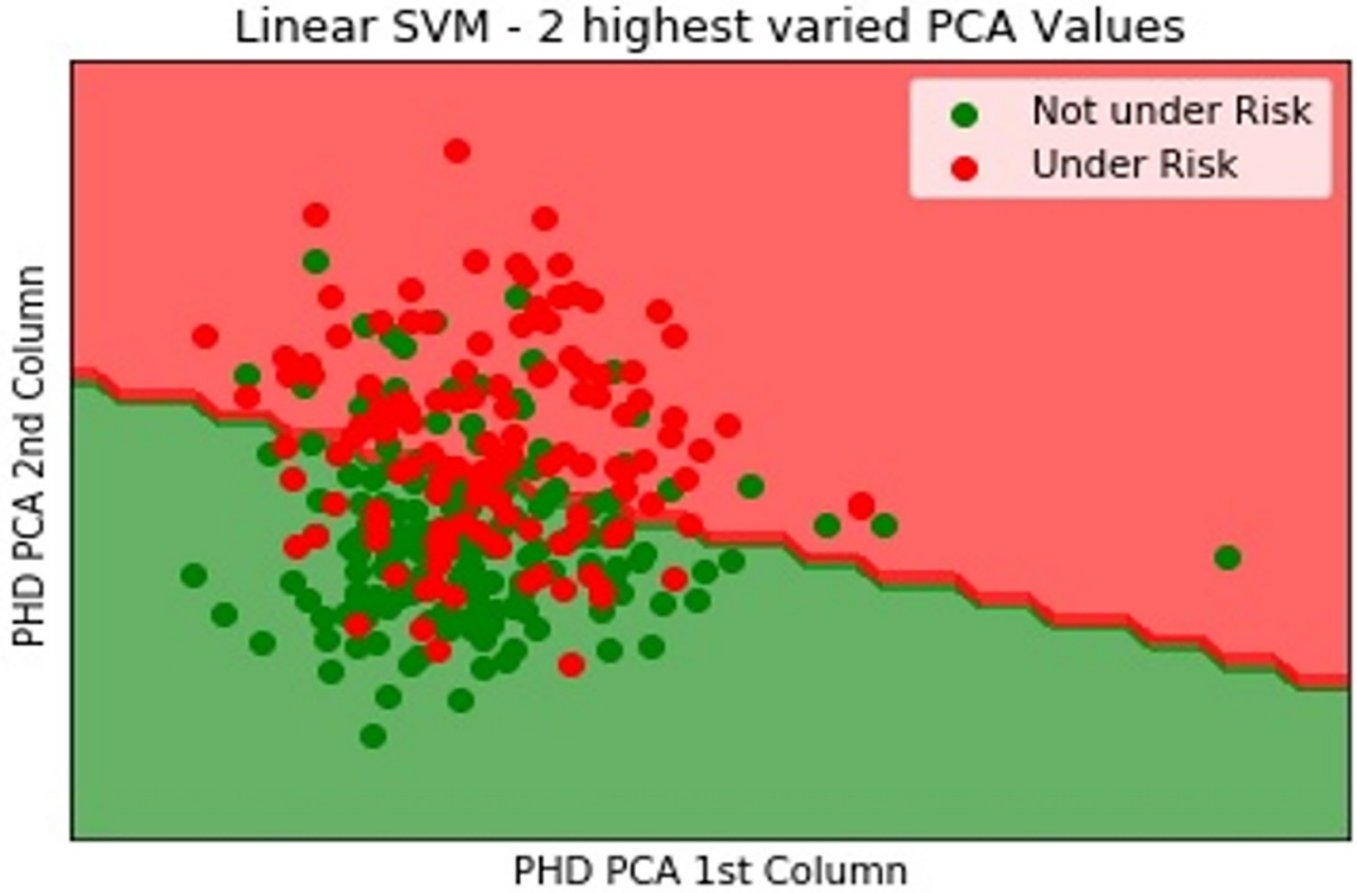
PHD classification using linear SVM.

In Fig 8, the data are further classified to the highest varied two principal component analysis (PCA) values using the linear SVM to display not-under-risk and under-risk data.

Here, red shows the under-risk heart disease data, and green shows the not-under-risk data. The linear support vector classification (SVC) values are split with two PCA values. The dataset is divided into 70% and 30% for training and testing, respectively. Subsequently, the training and test data were cross validated. To verify the accuracy of the fuzzy classified healthcare data, we used the linear SVM [61]. Although the previous classification accuracy of the used dataset is 79.4333%, in our case, the linear SVC values with the split are 0.8765213110411, i.e., 87% for healthcare data; the linear SVC score without a split is 0.8354674540625, i.e., 83%. The sensitivity of the model for the dataset using the SVM is 82.61%. The specificity of the model with healthcare data using the SVM is 81.63%. Similarly, the PPV for our model is 66.41% and the NPV for our model using the SVM in the healthcare dataset is 79.47%. It is necessary to remove the missing values and outliers and then fill the values with a mean data value. The missing values are removed using a Kalman filter. Fig 8 shows the linear SVC values with a split. The two PCA values are considered to show the highest variation in the classified health data.

**Fig 9:**
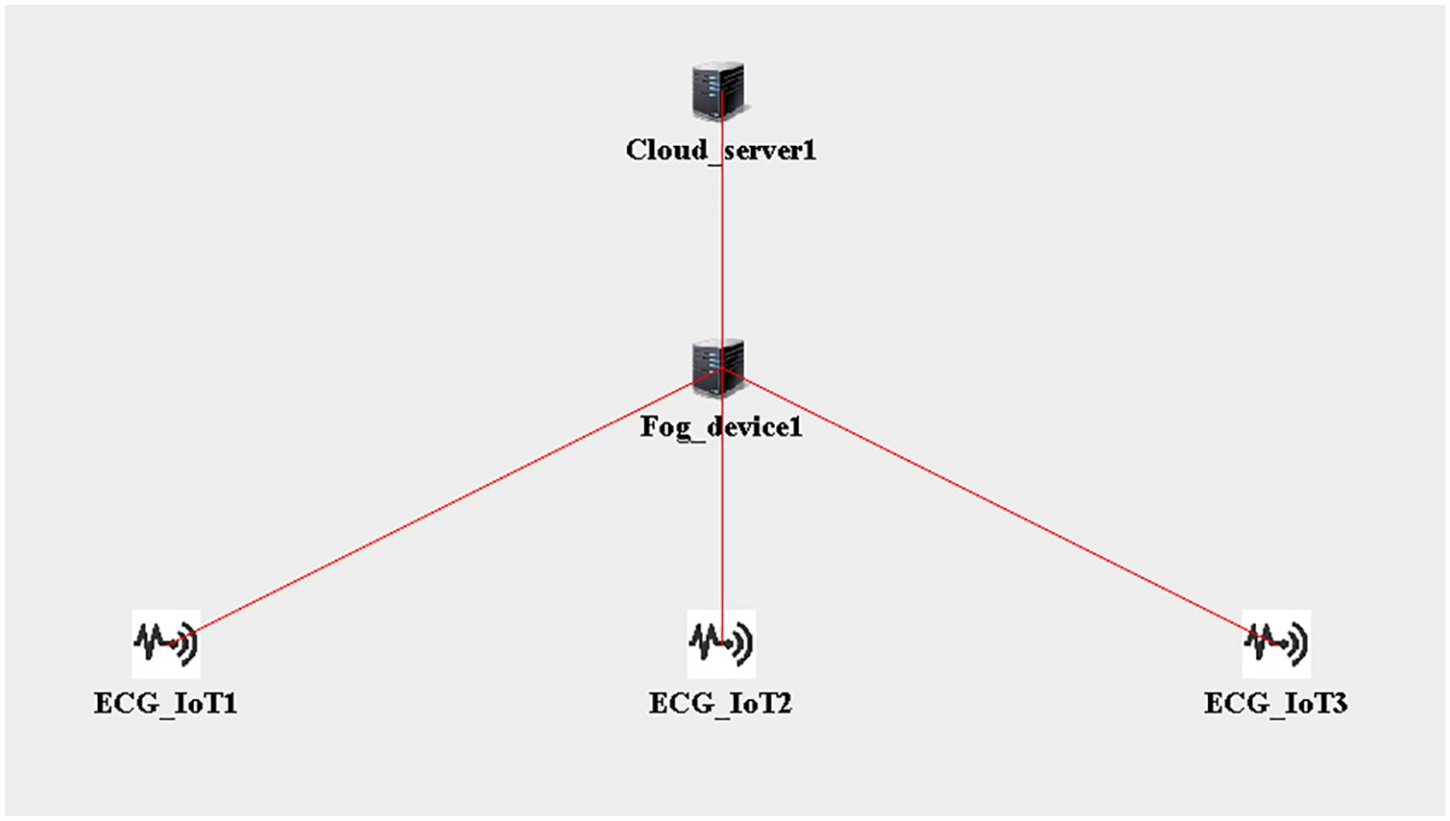
A graphical user interface (GUI) to build physical topology arrangements.

Fig 9 shows the GUI built on the iFogSim simulator. The simulation was performed to evaluate the latency, network consumption, and RAM usage for healthcare IoTs with respect to data transmission and data computation in fog nodes and cloud servers. The iFogSim simulator is based on the CloudSim simulator; it is used to simulate fog nodes and the healthcare IoT framework. Using the GUI in the simulator, we created physical elements such as fog devices, sensors, tuples, and connected links [14]. The physical topologies were built using the GUI and programmed using Java APIs. The object attributes were defined using the GUI in the topology. The topologies were stored and restored by modifying the topology from the JSON file format. Similar to the cloud, in iFogSim, IoTs and servers communicate with each other through message passaging and events. iFogSim enables the execution of multiple applications and supports the migration of application modules. The simulation performance was assessed based on various topology sizes. We used different libraries in iFogSim to execute the simulation. The simulation was conducted on an Intel^®^ i-7 core processor 4.30 GHz with 8 GB of RAM. The simulation involves a fog device to exchange data packets between the system entities. The simulation was performed for 3 h. The results demonstrated how different workloads and data allocation affected the latency. The fog device was connected to IoTs through Wi-Fi. To test the proposed algorithm performance in the iFogSim simulator, we varied the topology sizes by varying the IoT devices and maintaining the number of fog devices. The physical topology in iFogSim includes fog devices, ECG sensors, and cloud servers. Five configurations of the physical topology are simulated as config.1, config.2, config.3, config.4, and config.5. The new proposed algorithm was then programmed into the available libraries of the iFogSim simulator. This was performed to analyze the performance of the proposed algorithm using FC. Tables 6–9 show the descriptions of the fog devices, edge module, ECG sensor configuration, and network link. The data size for the PHD was defined in terms of megabytes.

**Table 6:** The description of fog device.

Device Type	CPU (GHz)	RAM (GB)
Fog_device1(Mobile device)	2.6	2
Cloud_server1(Cloud virtual machine)	4	4

**Table 7:** The description of the edge module. The CPU length (processing capacity) is in million instruction per second (MIPS).

Tuple types	CPU length (MIPS)	Network length (bytes)
Raw data (ECG) stream	1200	2100
PHD stream	2200	1700
Time-sensitive data stream	2800	1700

**Table 8:** ECG sensor configuration in iFogSim simulator.

CPU length	Network length (bytes)	Data packet average arrival time at different intervals (ms)
1200 million instructions	22000 bytes	25 milliseconds

**Table 9:** The network links description.

Source	Destination	Latency (ms)
ECG_IoT1	Fog_device1	40
ECG_IoT2	Fog_device1	45
ECG_IoT3	Fog_device1	45
Fog_device1	Cloud_server1	70

**Fig 10:**
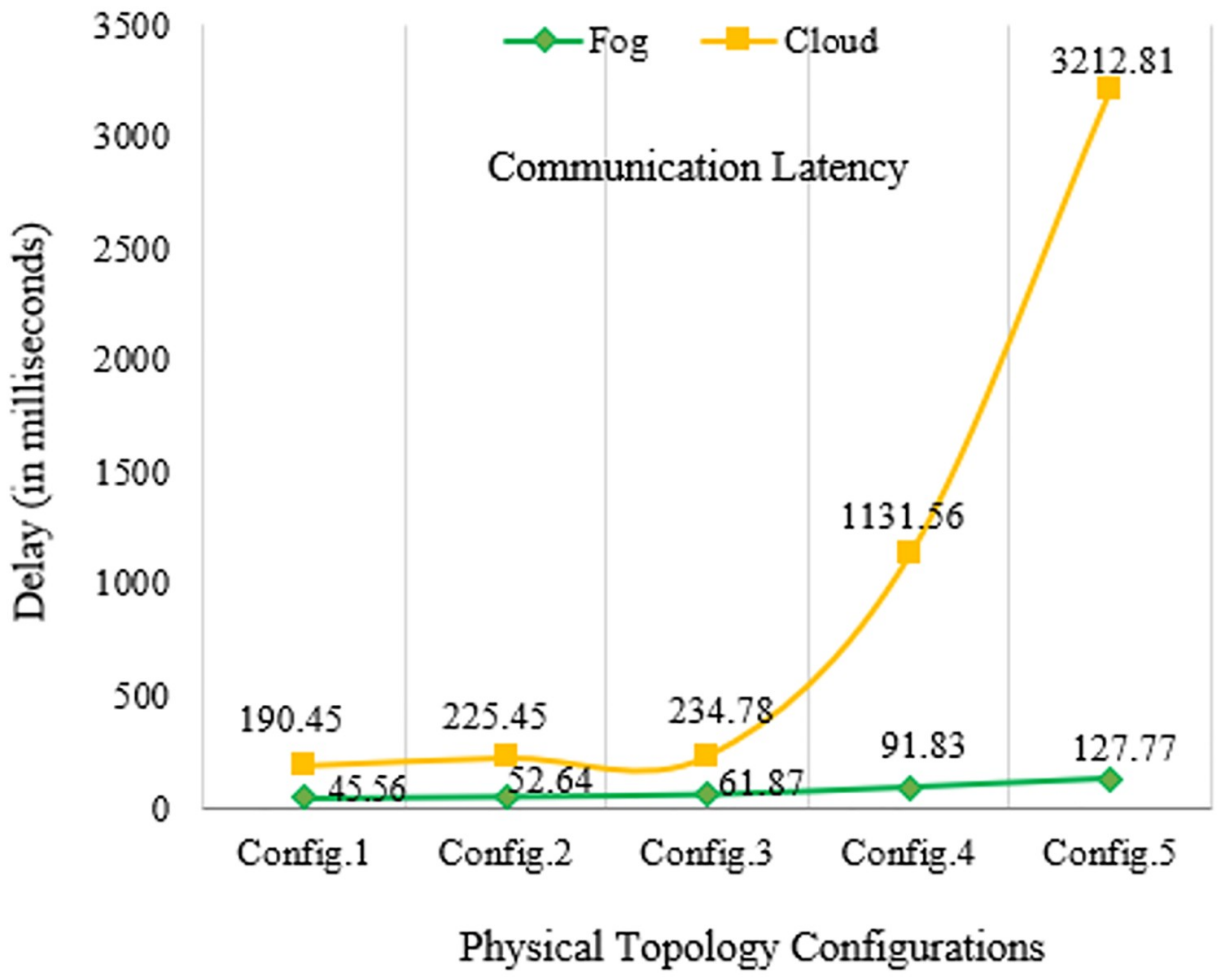
Communication latency comparison between FC and cloud computing.

**Fig 11:**
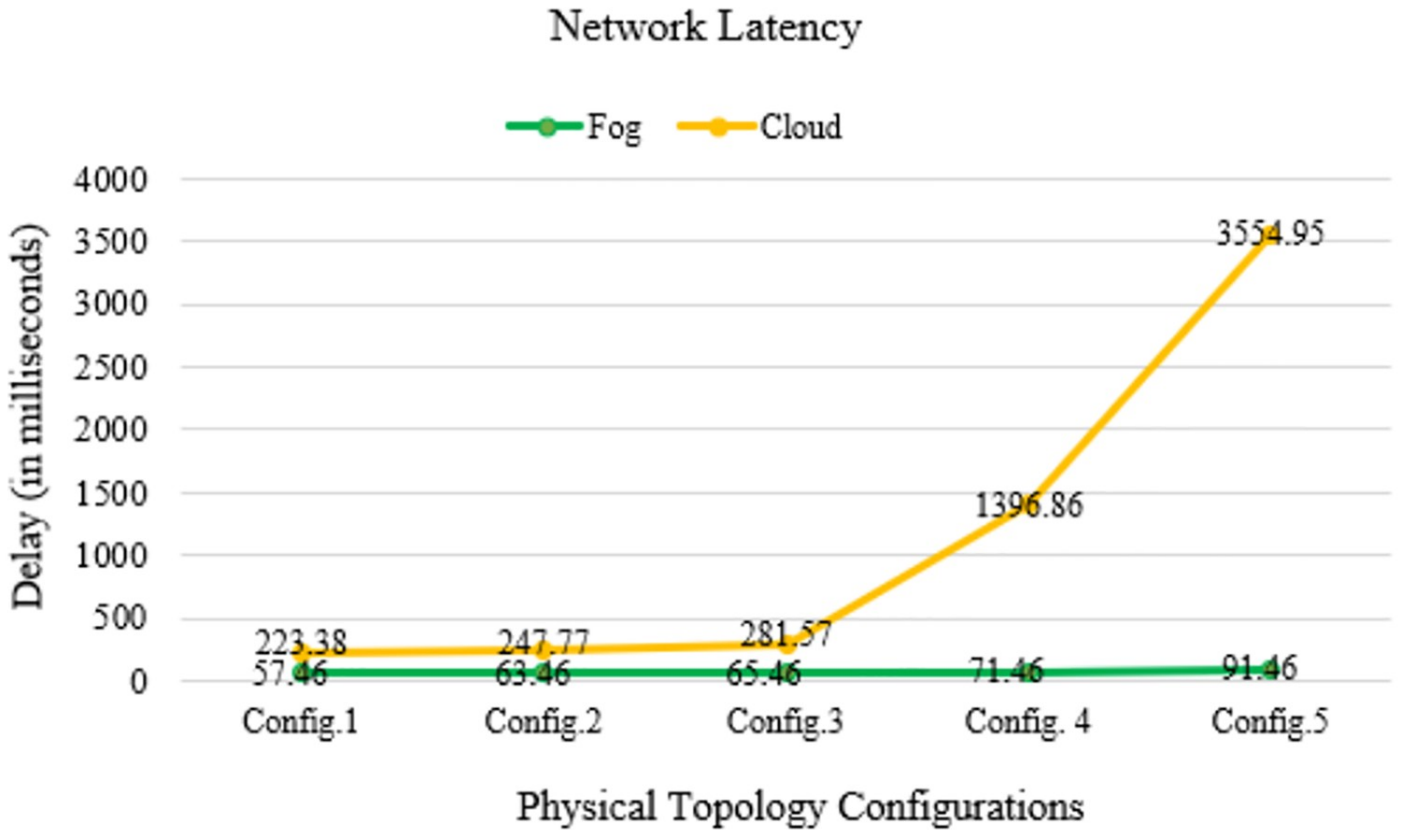
Network latency comparison between FC and cloud computing in IoT infrastructure.

**Fig 12:**
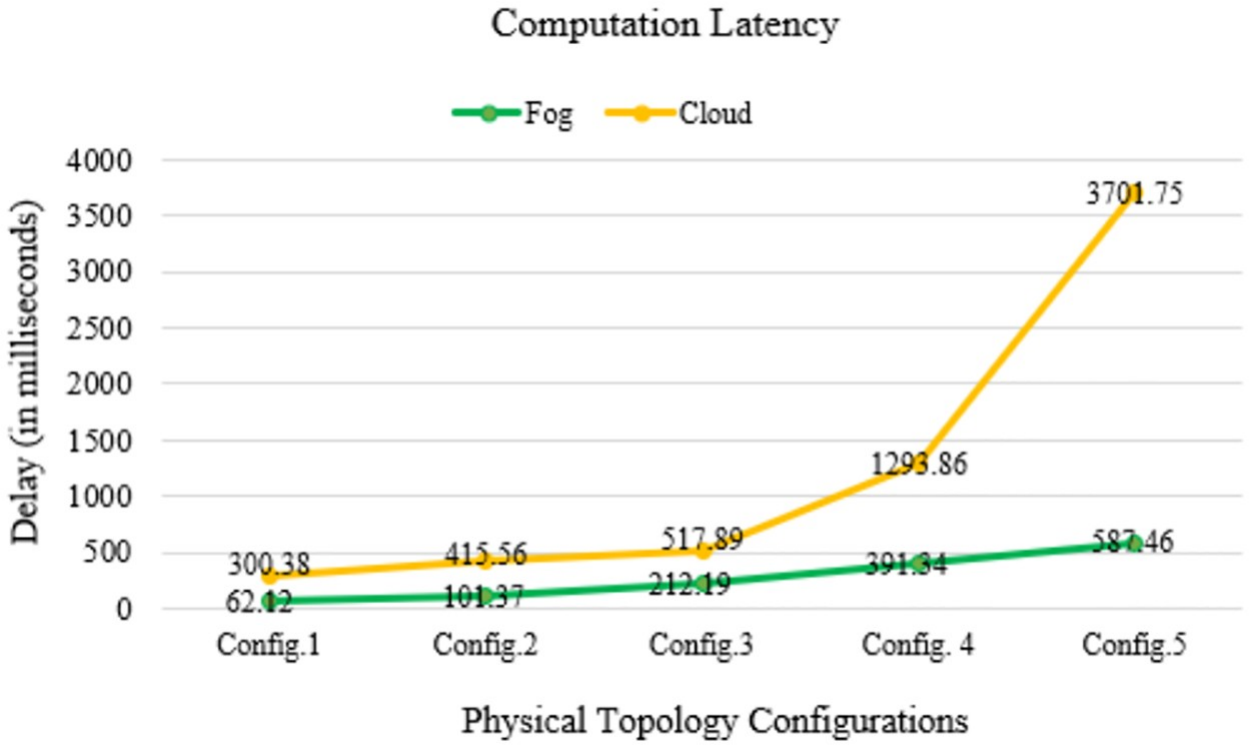
Computation latency comparison between FC and cloud computing.

**Fig 13:**
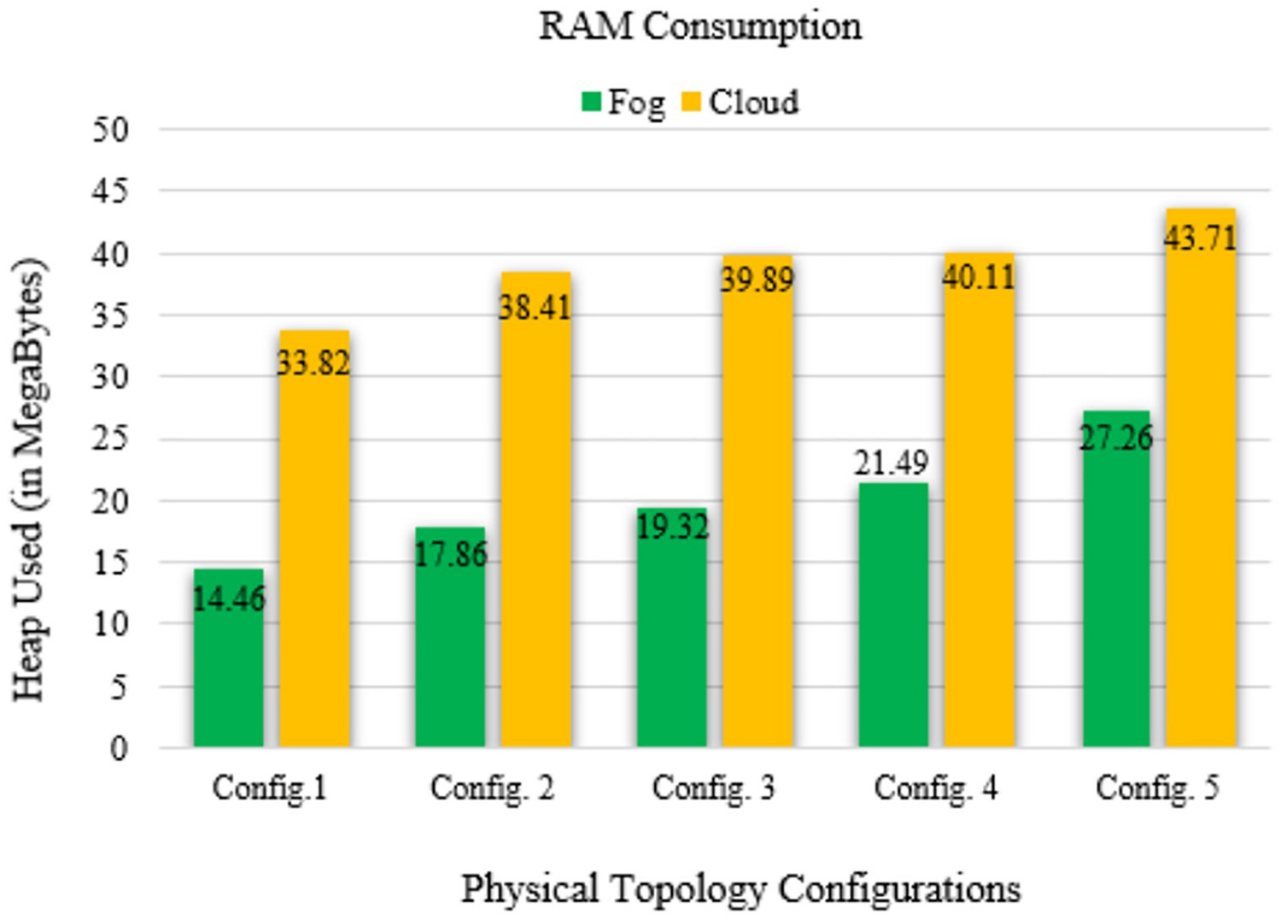
RAM consumption in FC and cloud computing.

**Fig 14:**
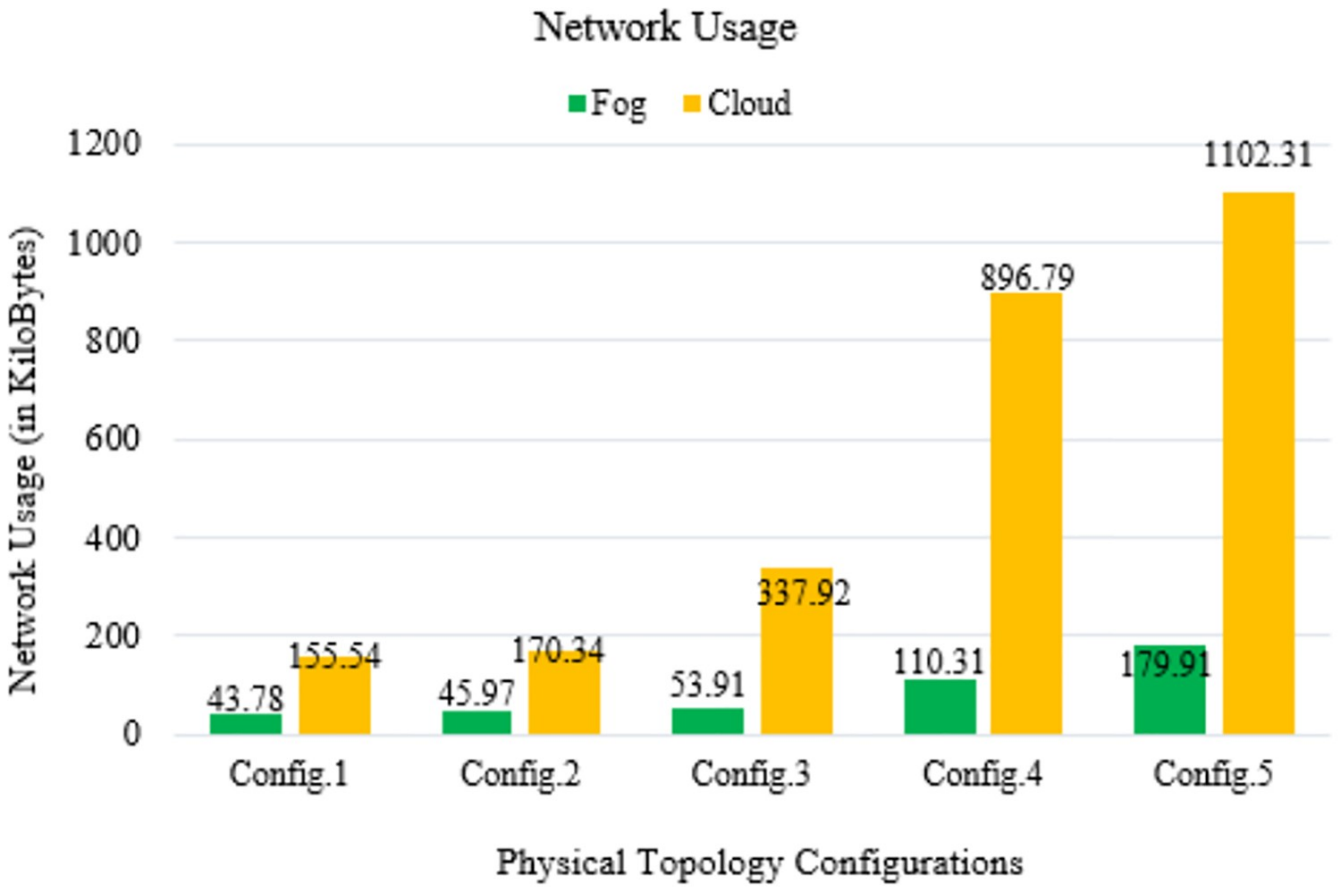
Network usage in FC and cloud computing.

In Figs 10–14 below, config.1, config.2, config.3, config.4, and config.5 show different physical topology configurations for FC and cloud computing in an IoT infrastructure.

Fig 10 shows a comparison of communication latency between FC and cloud computing under different topology configurations. In this simulation, an ECG sensor first generates a tuple (link) and sends it to the associated fog nodes, routers, and gateways. Once the tuple reaches the fog node, the fog server processes the incoming packet and sends the packet to another fog node. The fog node further sends the data packet to end-users.

The number of hop counts decreases when the data packets are transmitted between IoTs and fog computing servers. The algorithm implementation in iFogSim minimizes the network latency by distributing the fog nodes at the network edges. Meanwhile, the network latency increases when large data transmission occurs between IoTs and cloud servers.

Fig 12 shows the difference in computation latency between FC and cloud computing.

To measure the heap allocation, a massive heap profiler was used during the simulation of different topology sizes and input workloads. Here, the heap allocation did not escalate sufficiently with the increases in workload and physical topology configuration and size. The simulation scales with the minimum memory overhead despite an increase in the data transmission. Fig 13 shows the RAM consumption for data packet allocation and processing in fog nodes and cloud servers. The data packets are allocated at the edge of networks, thereby reducing the network usage. RAM is used with the input workload to quantify the heap allocation while simulations of different topology sizes and input workloads are performed.

Fig 14 shows the network usage by the ECG sensor device for data packet transmission in different physical topology configurations. Examining the fog devices, the network management degraded significantly as the fog nodes are distributed over a region. The results can also be interpreted as a fog-based scalable demonstration. Whereas in the case of cloud-based executions, the uncontrolled growth of networks results in network congestion and performance degradation. A fog-based deployment can be adopted for better efficiency and performance.

## Discussion

In our simulation, the average values of communication latency, network latency, and computation latency for the healthcare IoT infrastructure using FC in different physical topology configurations are 75.934, 69.86, and 270.896 ms, respectively. Furthermore, the average values of RAM consumption and network usage are minimized to 20.078 MB and 86.776 KB, respectively. Our proposed algorithm shows a minimization of latency percentage by 97–98% over other existing state-of-the-art methods. The results generated by simulating our proposed model demonstrated a better performance and efficiency in latency minimization compared with other known methods such as that of Hermes [28], which demonstrated an improvement in latency minimization by 16%; and Hipster [20], which demonstrated an 80–90% improvement in QoS for web searching. Another method, iFogStor [29], reduces the latency by more than 86% compared with cloud computing. The simulation outputs of our experiment were used to verify the execution gain of the prospective method. Additionally, the algorithm has low computational complexity. The results demonstrated that the RL method is compatible with the FC system. In this study, we modeled FC using RL and an NN. Some important parameters used in the simulation are summarized in Tables 6–9. The proposed work significantly reduced computational latency, communication latency, and network latency, as well as network usage and RAM consumption for healthcare IoTs. The experimental results demonstrated an enhanced execution of the proposed approach for latency minimization using FC.

## Conclusion

Healthcare IoT devices generate a large volume of data. Processing this leads to delay in services provided to end-users in an IoT-cloud environment. Traditional cloud services are unable to fulfill the latency demands of healthcare IoTs. Therefore, to minimize the high latency between healthcare IoTs, end-users, and cloud servers, we presented a FC-based analytical model. This model consists of fog nodes, fog servers, and the master fog controller, where end-users and patients can directly communicate to fog nodes in a single hop count. Then, we proposed a novel hybrid fuzzy-based RL algorithm employing NN evolutions strategies. The proposed algorithm was used for healthcare IoT data packet allocation and selection in a FC environment. The healthcare IoT data are classified using FIS and the linear SVM. The data packet allocation and selection are implemented using RL and NN evolution strategies in fog nodes. The issue of high latency was investigated using the following parameters: communication latency (ms), computation latency (ms), network latency (ms), network usage (KB), and RAM consumption (MB). The simulation of the proposed algorithm showed better results compared to those of existing techniques. Therefore, the proposed approach was concluded to be an optimal method, which indicates its applicability in healthcare IoTs. The proposed algorithm significantly reduces the high latency between healthcare IoTs and cloud servers. In the future, we plan to research the reliability and security of the healthcare IoT data using different cryptographic operations and techniques.

## Supporting information

S1 File Input healthcare ECG data. (XLS)

## References

[1] Hammi B, Khatoun R, Zeadally S, Fayad A, Khoukhi L. IoT technologies for smart cities. IET Networks. 2017;7(1):1–13.

[2] Wortmann F, Flüchter K. Internet of things. Business & Information Systems Engineering. 2015;57(3):221–4.

[3] Mukherjee M, Shu L, Wang D. Survey of fog computing: Fundamental, network applications, and research challenges. IEEE Communications Surveys & Tutorials. 2018;20(3):1826–57.

[4] Naha RK, Garg S, Georgakopoulos D, Jayaraman PP, Gao L, Xiang Y, et al Fog Computing: Survey of trends, architectures, requirements, and research directions. IEEE access. 2018;6:47980–8009.

[5] Nandyala CS, Kim H-K. From cloud to fog and IoT-based real-time U-healthcare monitoring for smart homes and hospitals. International Journal of Smart Home. 2016;10(2):187–96.

[6] Hassan MM, Lin K, Yue X, Wan J. A multimedia healthcare data sharing approach through cloud-based body area network. Future Generation Computer Systems. 2017;66:48–58.

[7] Meng X, Wang W, Zhang Z. Delay-constrained hybrid computation offloading with cloud and fog computing. IEEE Access. 2017;5:21355–67.

[8] Cirani S, Ferrari G, Iotti N, Picone M, editors. The IoT hub: A fog node for seamless management of heterogeneous connected smart objects. 2015 12th Annual IEEE International Conference on Sensing, Communication, and Networking-Workshops (SECON Workshops); 2015: IEEE.

[9] Gia TN, Jiang M, Rahmani A-M, Westerlund T, Liljeberg P, Tenhunen H, editors. Fog computing in healthcare internet of things: A case study on ecg feature extraction. 2015 IEEE International Conference on Computer and Information Technology; Ubiquitous Computing and Communications; Dependable, Autonomic and Secure Computing; Pervasive Intelligence and Computing; 2015: IEEE.

[10] Shi Y, Ding G, Wang H, Roman HE, Lu S, editors. The fog computing service for healthcare. 2015 2nd International Symposium on Future Information and Communication Technologies for Ubiquitous HealthCare (Ubi-HealthTech); 2015: IEEE.

[11] Rahmani AM, Gia TN, Negash B, Anzanpour A, Azimi I, Jiang M, et al Exploiting smart e-Health gateways at the edge of healthcare Internet-of-Things: A fog computing approach. Future Generation Computer Systems. 2018;78:641–58.

[12] Hung S-C, Liau D, Lien S-Y, Chen K-C, editors. Low latency communication for Internet of Things. 2015 IEEE/CIC International Conference on Communications in China (ICCC); 2015: IEEE.

[13] Lee G, Saad W, Bennis M. An online optimization framework for distributed fog network formation with minimal latency. IEEE Transactions on Wireless Communications. 2019.

[14] Gupta H, Vahid Dastjerdi A, Ghosh SK, Buyya R. iFogSim: A toolkit for modeling and simulation of resource management techniques in the Internet of Things, Edge and Fog computing environments. Software: Practice and Experience. 2017;47(9):1275–96.

[15] Skorin-Kapov L, Matijasevic M. Analysis of QoS requirements for e-health services and mapping to evolved packet system QoS classes. International journal of telemedicine and applications. 2010;2010:9. 10.1155/2010/628086 PMC2952804 20976301

[16] Gállego JR, Hernández-Solana Á, Canales M, Lafuente J, Valdovinos A, Fernández-Navajas J. Performance analysis of multiplexed medical data transmission for mobile emergency care over the UMTS channel. IEEE transactions on information technology in biomedicine. 2005;9(1):13–22. 15787003 10.1109/titb.2004.838362

[17] Bonomi F, Milito R, Zhu J, Addepalli S, editors. Fog computing and its role in the internet of things. Proceedings of the first edition of the MCC workshop on Mobile cloud computing; 2012: ACM.

[18] Liu Y, Fieldsend JE, Min G. A framework of fog computing: Architecture, challenges, and optimization. IEEE Access. 2017;5:25445–54.

[19] Deng R, Lu R, Lai C, Luan TH, Liang H. Optimal workload allocation in fog-cloud computing toward balanced delay and power consumption. IEEE Internet of Things Journal. 2016;3(6):1171–81.

[20] Nishtala R, Carpenter P, Petrucci V, Martorell X, editors. Hipster: Hybrid task manager for latency-critical cloud workloads. 2017 IEEE International Symposium on High Performance Computer Architecture (HPCA); 2017: IEEE.

[21] Conti S, Faraci G, Nicolosi R, Rizzo SA, Schembra G. Battery management in a green fog-computing node: a reinforcement-learning approach. IEEE Access. 2017;5:21126–38.

[22] Linthicum D. cisco 2018 [cited 2018 29 March]. https://blogs.cisco.com/perspectives/iot-from-cloud-to-fog.com.

[23] Alam MGR, Tun YK, Hong CS, editors. Multi-agent and reinforcement learning based code offloading in mobile fog. 2016 International Conference on Information Networking (ICOIN); 2016: IEEE.

[24] Baccarelli E, Naranjo PGV, Scarpiniti M, Shojafar M, Abawajy JH. Fog of everything: Energy-efficient networked computing architectures, research challenges, and a case study. IEEE access. 2017;5:9882–910.

[25] Wu J, Dong M, Ota K, Li J, Guan Z. FCSS: Fog computing based content-aware filtering for security services in information centric social networks. IEEE Transactions on Emerging Topics in computing. 2017.

[26] Dinh N-T, Kim Y. An Efficient Availability Guaranteed Deployment Scheme for IoT Service Chains over Fog-Core Cloud Networks. Sensors. 2018;18(11):3970. 10.3390/s18113970 PMC6263923 30445782

[27] Li G, Wu J, Li J, Wang K, Ye T. Service popularity-based smart resources partitioning for fog computing-enabled industrial Internet of Things. IEEE Transactions on Industrial Informatics. 2018;14(10):4702–11.

[28] Kao Y-H, Krishnamachari B, Ra M-R, Bai F. Hermes: Latency optimal task assignment for resource-constrained mobile computing. IEEE Transactions on Mobile Computing. 2017;16(11):3056–69.

[29] Naas MI, Parvedy PR, Boukhobza J, Lemarchand L, editors. iFogStor: an IoT data placement strategy for fog infrastructure. 2017 IEEE 1st International Conference on Fog and Edge Computing (ICFEC); 2017: IEEE.

[30] Pan J, McElhannon J. Future edge cloud and edge computing for internet of things applications. IEEE Internet of Things Journal. 2017;5(1):439–49.

[31] Cao H, Cai J. Distributed multiuser computation offloading for cloudlet-based mobile cloud computing: A game-theoretic machine learning approach. IEEE Transactions on Vehicular Technology. 2017;67(1):752–64.

[32] Brogi A, Forti S. QoS-aware deployment of IoT applications through the fog. IEEE Internet of Things Journal. 2017;4(5):1185–92.

[33] Mahmud R, Koch FL, Buyya R, editors. Cloud-fog interoperability in IoT-enabled healthcare solutions. Proceedings of the 19th International Conference on Distributed Computing and Networking; 2018: ACM.

[34] Rafique H, Shah MA, Islam SU, Maqsood T, Khan S, Maple C. A Novel Bio-Inspired Hybrid Algorithm (NBIHA) for Efficient Resource Management in Fog Computing. IEEE Access. 2019;7:115760–73.

[35] Ahsan MM, Ali I, Imran M, Idris MYI, Khan S, Khan A. A Fog-centric Secure Cloud Storage Scheme. IEEE Transactions on Sustainable Computing. 2019.

[36] Waqar A, Raza A, Abbas H, Khan MK. A framework for preservation of cloud users’ data privacy using dynamic reconstruction of metadata. Journal of Network and Computer Applications. 2013;36(1):235–48.

[37] Soleymani SA, Abdullah AH, Zareei M, Anisi MH, Vargas-Rosales C, Khan MK, et al A secure trust model based on fuzzy logic in vehicular ad hoc networks with fog computing. IEEE Access. 2017;5:15619–29.

[38] Andras Janosi WS, Matthias Pfisterer, Robert Detrano. UCI Machine Learning Repository 2018 [cited 2018 25 February]. https://archive.ics.uci.edu/ml/datasets/heart+Disease.

[39] Aha D, Kibler D. Instance-based prediction of heart-disease presence with the Cleveland database. University of California. 1988;3(1):3.2.

[40] Blake CL, Merz CJ. UCI repository of machine learning databases, 1998. 1998.

[41] Detrano R, Janosi A, Steinbrunn W, Pfisterer M, Schmid J-J, Sandhu S, et al International application of a new probability algorithm for the diagnosis of coronary artery disease. The American journal of cardiology. 1989;64(5):304–10. 10.1016/0002-9149(89)90524-9 2756873

[42] Gennari JH, Langley P, Fisher D. Models of incremental concept formation. Artificial intelligence. 1989;40(1–3):11–61.

[43] Le TP, Vien NA, Chung T. A deep hierarchical reinforcement learning algorithm in partially observable Markov decision processes. IEEE Access. 2018;6:49089–102.

[44] Mai L, Dao N-N, Park M. Real-Time Task Assignment Approach Leveraging Reinforcement Learning with Evolution Strategies for Long-Term Latency Minimization in Fog Computing. Sensors. 2018;18(9):2830. 10.3390/s18092830 PMC6163362 30150577

[45] Aazam M, Huh E-N, editors. Fog computing and smart gateway based communication for cloud of things. 2014 International Conference on Future Internet of Things and Cloud; 2014: IEEE.

[46] Baek J-y, Kaddoum G, Garg S, Kaur K, Gravel V. Managing Fog Networks using Reinforcement Learning Based Load Balancing Algorithm. arXiv preprint arXiv:190110023. 2019.

[47] Sundharakumar K, Dhivya S, Mohanavalli S, Chander RV. Cloud based fuzzy healthcare system. Procedia computer science. 2015;50:143–8.

[48] Kaur P, Khurmi DSS, Josan DGS. Fuzzy based analysis of proposed model for physical health standard based on association rule mining techniques. International Journal of Computer Science and Communication Engineering. 2012;1(2).

[49] Kraemer FA, Braten AE, Tamkittikhun N, Palma D. Fog computing in healthcare–a review and discussion. IEEE Access. 2017;5:9206–22.

[50] Osanaiye O, Chen S, Yan Z, Lu R, Choo K-KR, Dlodlo M. From cloud to fog computing: A review and a conceptual live VM migration framework. IEEE Access. 2017;5:8284–300.

[51] Yousefpour A, Ishigaki G, Jue JP, editors. Fog computing: Towards minimizing delay in the internet of things. 2017 IEEE International Conference on Edge Computing (EDGE); 2017: IEEE.

[52] Rolim CO, Koch FL, Westphall CB, Werner J, Fracalossi A, Salvador GS, editors. A cloud computing solution for patient’s data collection in health care institutions. 2010 Second International Conference on eHealth, Telemedicine, and Social Medicine; 2010: IEEE.

[53] Shahid N, Rappon T, Berta W. Applications of artificial neural networks in health care organizational decision-making: A scoping review. PloS one. 2019;14(2):e0212356 10.1371/journal.pone.0212356 30779785 PMC6380578

[54] Wang M, Lu S, Zhu D, Lin J, Wang Z, editors. A High-Speed and Low-Complexity Architecture for Softmax Function in Deep Learning. 2018 IEEE Asia Pacific Conference on Circuits and Systems (APCCAS); 2018: IEEE.

[55] Michalewicz Z. Evolution strategies and other methods Genetic algorithms+ data structures = evolution programs: Springer; 1994 p. 167–84.

[56] Verma S, Yadav AK, Motwani D, Raw R, Singh HK, editors. An efficient data replication and load balancing technique for fog computing environment. 2016 3rd International Conference on Computing for Sustainable Global Development (INDIACom); 2016: IEEE.

[57] L’heureux A, Grolinger K, Elyamany HF, Capretz MA. Machine learning with big data: Challenges and approaches. IEEE Access. 2017;5:7776–97.

[58] Fu Xin Y H, Chenxi Liu, Wang Jianwei, Wang Yinhai. A hybrid neural network for large-scale expressway network OD prediction based on toll data. PloS one. 2019:15. 10.1371/journal.pone.0217241 PMC6532916 31120962

[59] Saqib E, Awan MB, Sohel Ferdous, Sanfilippo Frank M., Chow Benjamin J., Dwivedi Grish. Feature selection and transformation by machine learning reduce variable numbers and improve prediction for heart failure readmission or death. PloS one. 2019:13. 10.1371/journal.pone.0218760 PMC6594617 31242238

[60] Sebban M, Nock R, Lallich S. Stopping criterion for boosting-based data reduction techniques: from binary to multiclass problem. Journal of machine learning research. 2002;3(Dec):863–85.

[61] Ehteram M, Singh VP, Ferdowsi A, Mousavi SF, Farzin S, Karami H, et al An improved model based on the support vector machine and cuckoo algorithm for simulating reference evapotranspiration. PloS one. 2019;14(5):e0217499 10.1371/journal.pone.0217499 31150443 PMC6544354

